# LncRNA BACE1-AS delays the propagation of *Cryptosporidium parvum* through regulating cell apoptosis by targeting the miR-6805-5p/IRF3 axis

**DOI:** 10.1128/spectrum.02022-24

**Published:** 2025-06-09

**Authors:** Shuang Huang, Xin Yang, Ting-Li Liu, Gui-Rong Hu, Qian Yao, Jun-Ke Song, Ying-Ying Fan, Guang-Hui Zhao

**Affiliations:** 1 College of Veterinary Medicine, Northwest A&F University 12469, Yangling, China; University of Illinois Urbana-Champaign, Urbana, Illinois, USA

**Keywords:** *Cryptosporidium parvum*, BACE1-AS, miR-6805-5p, IRF3, apoptosis, propagation

## Abstract

*Cryptosporidium* spp. are important zoonotic pathogens causing diarrhea in humans and various animals. To date, there are still no effective drugs and vaccines for the prevention and treatment of cryptosporidiosis, mainly due to the absence of knowledge on the interaction mechanisms between hosts and *Cryptosporidium*. Increasing evidence has indicated that long non-coding RNAs (lncRNAs) play significant roles in various physiological and pathological processes. Our previous study showed that *Cryptosporidium parvum* infection induced significant differential changes in lncRNA expression profiles in HCT-8 cells. BACE1-AS was a significantly downregulated lncRNA in HCT-8 cells during *C. parvum* infection, but its roles were still unknown. Results of the present study indicated that *C. parvum* infection significantly downregulated the expression of BACE1-AS probably through regulating the nuclear factor kappa-B (NF-κB) signaling pathway, and the expression of BACE1-AS was negatively related to the propagation of *C. parvum* in HCT-8 cells. BACE1-AS was mainly localized outside of the nucleus of HCT-8 cells and promoted the expression of *IRF3* by sponging miR-6805-5p during *C. parvum* infection. Further studies indicated that the BACE1-AS/miR-6805-5p/IRF3 axis delayed *in vitro* propagation of *C. parvum* through regulating BCL2-mediated apoptosis of HCT-8 cells. The present study enriches our knowledge on the function of host lncRNAs during *Cryptosporidium* infection and contributes to our understanding of the interaction between hosts and *Cryptosporidium*.

**IMPORTANCE:**

Recent studies indicate that *Cryptosporidium* can regulate cell apoptosis to promote its development in host cells, but the mechanism is still unclear. This study identified a significantly downregulated host lncRNA BACE1-AS during *C. parvum* infection, which could regulate cell apoptosis to affect the propagation of *C. parvum in vitro* by targeting the miR-6805-5p/IRF3 axis. The present study contributes to our understanding of the pathogenesis of *Cryptosporidium* and provides potential targets for the development of drugs and vaccines against cryptosporidiosis.

## INTRODUCTION

*Cryptosporidium* is an important zoonotic protozoan parasite causing diarrhea of humans and a great number of animals (e.g., cattle and goats) worldwide (1, 2). Cryptosporidiosis caused by *Cryptosporidium* infection can lead to severe or even life-threatening diarrhea in children and immunosuppressed populations, and *Cryptosporidium* has been recognized as one of the most important diarrheal pathogens in children under 2 years in low- and middle-income countries (3–6). In 2016, diarrhea related to *Cryptosporidium* infection caused over 48,000 deaths in children less than 5 years (1). However, there are still no effective vaccines or drugs for the prevention and treatment of cryptosporidiosis in humans and animals. Currently, only one drug, nitazoxanide, is approved by the Food and Drug Administration for the treatment of human cryptosporidiosis, but it has little effect on immunosuppressed individuals and children (7, 8). Understanding the interaction between *Cryptosporidium* and hosts contributes to the discovery of potential targets for the development of drugs and vaccines and thus provides a theoretical basis for the prevention and control of cryptosporidiosis.

Long non-coding RNAs (lncRNAs) are a class of RNAs with over 200 nucleotides in length, and increasing evidence suggests that lncRNAs play important roles in physiological activities (e.g., organ aging and embryogenesis) (9, 10) and pathological processes (e.g., cancer and neurodegenerative diseases) (11–14) by interacting with DNA, RNA, or proteins and can be potential markers for the diagnosis of cancers (15–18). Recent studies have shown that the lncRNA expression profiles in host cells can be altered following infection of parasites, such as *Cryptosporidium parvum* (19, 20). Moreover, aberrant expression of lncRNAs plays important roles in the development, propagation, and survival of parasites (21–23).

Previous studies have found that several significantly differently expressed lncRNAs (e.g., NR_045064, Nostrill, XR_001779380, and U90926) in murine intestinal epithelial cells (IEC4.1) following *C. parvum* infection can facilitate intestinal anti-*Cryptosporidium* defense by regulating transcription of defense genes (24–27). Considering the serious hazard of *C. parvum* to humans, our group explored the effect of *C. parvum* infection on the expression of lncRNAs in human ileocecal adenocarcinoma (HCT-8) cells and found that *C. parvum* infection could induce significant changes in the lncRNA expression profile in HCT-8 cells (19), but the roles of those significantly differently expressed lncRNAs in HCT-8 cells during *C. parvum* infection were seldomly explored. The present study aimed to apply an *in vitro* model of HCT-8 cells infected with *C. parvum* to explore the effect and mechanism of BACE1-AS, a significantly downregulated lncRNA following *C. parvum* infection, in regulating the propagation of *C. parvum in vitro*, and the findings can enrich our knowledge on the interaction between hosts and *Cryptosporidium*.

## MATERIALS AND METHODS

### Parasites, cell culture and *in vitro* infection model

The *C. parvum* IIdA19G1 oocysts used in the present study were previously preserved in our lab and passaged in a newborn calf every 3 months as reported (28). *C. parvum* oocysts were cultured in HCT-8 cells to establish an *in vitro* infection model as previously reported in our lab (28). Meanwhile, tachyzoites of *Toxoplasma gondii* (RH strain) and *Neospora caninum* (NC-1 strain) preserved in our lab were used to establish an *in vitro* infection model in HCT-8 cells at a multiplicity of infection of 3:1 (parasite:cell) using the same method as *C. parvum*, respectively (28).

### Reverse transcription-quantitative real-time polymerase chain reaction **(RT-qPCR)**

Cell samples were treated with TRIzol reagent (Hunan Accurate Biology Co., Ltd., Hunan, China) to extract the total RNA, and the RNA sample was then reverse transcribed to cDNA as reported (28). The mRNA levels of microRNAs (miRNAs) and mRNAs were detected by using SYBR Green Mix (ABclonal, Wuhan, China) as previously reported (28), with human *U6* and *GAPDH* as internal controls, respectively. The relative mRNA levels were determined using the 2^−ΔΔCt^ method. Each experiment was repeated twice. Primer sequences of genes detected in this study were indicated in Table 1.

**TABLE 1:** Sequence information of the primers used for RT-qPCR in this study^*a*^

Primer names	Forward sequence (5′−3′)	Reverse sequence (5′−3′)
BACE1-AS	AAGCTTGGCTCACCGCAACCTCCACCGT	CTCGAGTTAAAAGCACTAAACAAGGTA
*IRF3*	TAAGCTTATGGGAACCCCAAAGCC	ATCTCGAGTCAGCTCTCCCCAGGG
*CDX2*	AAGGAGTTTCACTACAGTCGC	GGACACTTCTCAGAGGACCT
*GAS7*	TCAACACGACCACCAATG	GAAGCCAAGGAGTTCTGAG
*TNFSF13*	CTGCTGACCCAACAAACA	TTGCCACATCACCTCTGTC
*MAPK2*	TGAAGACACAACACCTCAGC	GGAGTCAGCATTTGGGAA
*HMGA1*	AAAGGACGGCACTGAGAA	CTTCCTCCTTCTCCAGTT
*IRF9*	ATGTTGCTGAGCCCTACA	CCCTCCTCCTCATTATTG
*CEP164*	GCCTTGGGTTCCTCATTA	TCCTCATCCTCCTCATTG
*ATG12*	TGACCTGCTGGCTGAATACCT	GATGTGAAACCAAAACGCCTAAC
*PTEN*	ACTATTCCCAGTCAGAGGCG	ACTTGTCTTCCCGTCGTGT
*hsp70*	AACTTTAGCTCCAGTTGAGAAAGTACTC	CATGGCTCTTTACCGTTAAAGAATTCC
*GAPDH*	GAACATCATCCCTGCCTCT	CCTGCTTCACCACCTTCTT
miR-6805-5p	ATTATTAGGGGGCGGCTTGT	Universal primer
miR-541-3p	GTGGGCACAGAATCTGGACT	Universal primer
miR-143-3p	GCTGAGATGAAGCACTGTAGCTC	Universal primer
miR-762	TTAACATATGGGGCTGGGGCC	Universal primer
miR-5088-3p	GCTGATTGTAGCCTTTTGGAGTAGA	Universal primer
miR-654-3p	TGGTGGGCACAGAATCTGGA	Universal primer
miR-12120-3p	TAATTATAAGGAACGCGGGGCCTT	Universal primer
miR-5582-5p	GGCGTAGGCACACTTAAAGTTATAGC	Universal primer
miR-6798-5p	TTATATACCAGGGGGATGGGCGA	Universal primer
miR-6803-5p	TATTATTATCTGGGGGTGGGGGGC	Universal primer
miR-6851-5p	TATTAGGAGGTGGTACTAGGGGCCA	Universal primer
miR-296-5p	TAAAGGGCCCCCCCTCAAT	Universal primer
*U6*	GGAACGATACAGAGAAGATTAGC	TGGAACGCTTCACGAATTTGCG

^*a*^ *ATG12*, *autophagy-related 12*; BACE1-AS, *antisense transcript of β-secretase 1*; *CDX2*, *caudal type homeobox 2*; *CEP164*, *centrosomal protein 164*; *GAPDH*, *glyceraldehyde-3-phosphate dehydrogenase*; *GAS7*, *growth arrest-specific 7*; *HMGA1*, *high mobility group AT-hook 1*; *hsp70*, *heat shock protein 70*; *IRF3*, *interferon regulatory factor 3*; *IRF9*, *interferon regulatory factor 9*; *MAPK2*, *mitogen-activated protein kinase 2*; *PTEN*, *phosphatase and tensin homolog*; *TNFSF13*, *TNF superfamily member 13*; *U6*, *RNU6-1*.

### Western blot

Western blot was utilized to analyze the protein expression levels of IRF3, BCL2, cleaved caspase-3, and BAX as reported (28). The primary antibodies used in this study included rabbit anti-IRF3 (1:2,000; Abways Technology, Shanghai, China), mouse anti-GAPDH (1:5,000; Abways Technology), rabbit anti-BCL2 (1:2,000; Abways Technology), rabbit anti-cleaved caspase-3 p17 (1:2,000; Abways Technology), and rabbit anti-BAX (1:2,000; Abways Technology). The secondary antibodies were horseradish peroxidase (HRP)-conjugated goat anti-rabbit (1:10,000; Sangon Biotech, Shanghai, China) and HRP-conjugated goat anti-mouse (1:10,000; Sangon Biotech). Protein bands were visualized and imaged by applying an enhanced chemiluminescence system (Applygen Technologies Inc., Beijing, China) and an automatic gel imaging analysis system (Sage, Beijing, China), respectively.

### Fluorescence *in situ* hybridization

Fluorescence *in situ* hybridization (FISH) was performed to identify the subcellular localization of BACE1-AS by using RNA FISH Probe Kit B (SA-Biotin System; GenePharma, Shanghai, China) according to the instructions. Briefly, cover glasses were placed into a 48-well plate, and then HCT-8 cells were seeded on the cover glasses at a density of 10^4^ cells/well and cultured under 5% CO_2_ at 37°C. The cells were washed with phosphate-buffered saline (PBS) and fixed with 4% paraformaldehyde at room temperature for 15 min when cell density reached 60%, permeated with 0.1% Triton X-100 at room temperature for 15 min, incubated with 1× sealing solution at 37°C for 30 min, and washed with 2× saline-sodium citrate (SSC) at 37°C for 30 min. Then, the probe mixtures prepared in advance were added to the cells for hybridization overnight at 37°C under dark conditions. Subsequently, the cells were washed with 0.1% Tween 20-added PBS three times, stained with 2-(4-amidinophenyl)-6-indolecarbamidine dihydrochloride (DAPI) (Beyotime Biotech, Shanghai, China) for 10–15 min, and washed with PBS three times. Finally, the cells were photographed under a confocal microscope (Leica, Wetzlar, Germany) after the addition of the antifluorescence quencher.

### Prediction of targeted miRNAs and mRNAs

LncBook (https://ngdc.cncb.ac.cn/lncbook/home) was used to predict the targeted miRNAs of BACE1-AS, and those highly matched miRNAs were selected for further RT-qPCR identification based on their site type (7mer-m8), score (>100), and energy (<−20). Meanwhile, miRwalk (http://129.206.7.150) was applied to predict the targeted mRNAs of miR-6805-5p, and those highly matched mRNAs were applied for further verification based on their site type (7mer-m8) and score percentile (>90).

### Cell transfection

Overexpression vectors of BACE1-AS [pcDNA3.1(+)-BACE1-AS] and *IRF3* [pcDNA3.1(+)-*IRF3*] were constructed by cloning the CDS regions of BACE1-AS and *IRF3* into pcDNA3.1(+) plasmid (Invitrogen, Gaithersburg, MD, USA), respectively, and verified by sequencing in Sangon Biotech. Small interfering RNAs (siRNAs) against BACE1-AS (si-BACE1-AS) and *IRF3* (si-*IRF3*), scramble RNA (si-control), miR-6805-5p mimics, control mimics, miR-6805-5p inhibitor, and control inhibitor were synthesized by GenePharma. Lipofectamine 2000 reagent (Invitrogen) was applied to transfect those plasmids, siRNAs, mimics, and inhibitors into cells according to the instruction. The sequences of si-BACE1-AS, si-*IRF3*, miR-6805-5p mimics, and inhibitor are indicated in Table S1. All the plasmids, siRNAs, mimics, and inhibitors used in the present study were verified by RT-qPCR and/or Western blot before further experiments (Fig. S1 and S2).

### Detection of the propagation of *C. parvum* in HCT-8 cells by both RT-qPCR and immunofluorescence assay

The propagation of *C. parvum* in HCT-8 cells was detected by both RT-qPCR and immunofluorescence assay (IFA). The mRNA levels of *C. parvum hsp70* were detected for the quantification of the propagation of *C. parvum* in HCT-8 cells by using RT-qPCR as abovementioned in section “RNA extraction and quantitative real-time polymerase chain reaction”, with human *GAPDH* as the internal control (28–30). Furthermore, IFA was used to analyze the propagation of *C. parvum in vitro* (28). Briefly, HCT-8 cells were inoculated on slides for further experiments. After the experiments, the cells were washed with pre-cooled PBS, fixed with methanol at 4°C for 15 min, permeabilized with 0.5% Triton X-100 at room temperature for 15 min, and blocked with 5% non-fat milk at room temperature for 1 h. The cells were incubated with bovine anti-*Cryptosporidium* serum (1:200, isolated from the blood of a calf artificially infected with *C. parvum*) overnight at 4°C, washed with PBS three times, and then incubated with fluorescein isothiocyanate (FITC)-conjugated goat-antibovine IgG (1:500, Sangon Biotech) at 4°C for 1 h. After being washed with PBS three times, the cells were stained with DAPI and photographed under a fluorescence microscope (Leica) after treatment with antibody quencher. The results were analyzed by using ImageJ v.1.4.3.67 (https://imagej.nih.gov/ij/), and 50 randomly visual fields were captured under ×200 microscope for quantitative analysis for each cover glass. The propagation efficiency of *C. parvum* was calculated by counting the average percentage of FITC-labeled *C. parvum* to DAPI-labeled HCT-8 cells in each microscope field (28). The procedure was repeated twice for each group. The relative propagation efficiency of *C. parvum* was analyzed by using the 2^−ΔΔCt^ method.

### Dual luciferase reporter assay

The dual luciferase reporter gene assay is a molecular biology technique that simultaneously monitors the activity of two genes by using two different luciferase enzymes, namely, Firefly Luciferase (F-Luc) and Renilla Luciferase (R-Luc). This technology provides an efficient and reliable method for studying gene expression regulation. The dual luciferase reporter gene experiment relies on two naturally occurring luciferase enzymes in organisms that can catalyze the luminescence of specific substrates. The pmirGLO vector is a plasmid that allows quantitative measurement of miRNA activity by inserting miRNA binding sites downstream of the F-Luc gene. In experiments, F-Luc is usually used as the main reporter gene to evaluate the activity of target promoters, while R-Luc serves as an internal control to help correct experimental variations such as cell number and transfection efficiency. By comparing the relative activity of two enzymes, researchers can obtain reliable information about gene expression regulation. In this study, the luciferase reporter plasmids of wild-type BACE1-AS (BACE1-AS -WT), mutant BACE1-AS (BACE1-AS -MUT), wild-type *IRF3* (*IRF3*-WT), and mutant *IRF3* (*IRF3*-MUT) were constructed by cloning BACE1-AS, the miR-6805-5p binding site-mutated BACE1-AS, 3′ UTR region of *IRF3*, and the miR-6805-5p binding site-mutated 3′ UTR region of *IRF3* into pmirGLO vectors (Promega, Madison, WI, USA), respectively. Each abovementioned recombinant plasmid was co-transfected with miR-6805-5p mimics or control mimics into HCT-8 cells by applying Lipofectamine 2000 (Thermo Fisher Scientific, Waltham, MA, USA). Luciferase activities were detected as previously reported (28).

### Cell apoptosis analysis

Cell apoptosis was measured by applying a commercial Annexin V-FITC/propidium iodide (PI) apoptosis detection kit (Yeasen, Shanghai, China) according to the instruction. Briefly, HCT-8 cells were cultured in six-well plates for 24 h, then digested with pancreatic enzyme without ethylene diamine tetraacetic acid (EDTA) for 5 min and centrifuged at 4°C for 5 min. Subsequently, the cells were washed twice with 1× PBS and resuspended with 200 µL 1× binding buffer. Finally, 5 µL Annexin V-FITC and 10 µL PI were successively added, mixed, and incubated for 15 min. The apoptosis level was detected and visualized for data analysis by using a digital flow cytometer (BD, New Jersey, USA).

### Statistical analysis

Differences between groups were analyzed by applying Student’s *t*-test or one-way analysis of variance test in IBM SPSS v.22.0 (Armonk, NY, USA). All data were indicated as mean ± standard deviation. Significant differences were reported if the *P* value was less than 0.05.

## RESULTS

### Downregulation of BACE1-AS is likely triggered by the NF-κB signaling pathway during *C. parvum* infection

**Fig 1:**
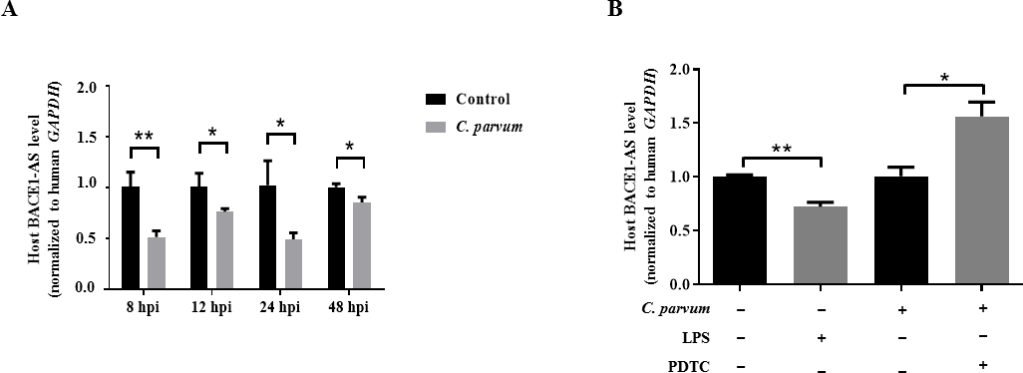
Expression of BACE1-AS in HCT-8 cells during *C. parvum* infection or LPS/PDTC stimulation. (A) The mRNA levels of BACE1-AS in HCT-8 cells infected with *C. parvum* from 8 to 48 hpi by using RT-qPCR. (B) The mRNA levels of BACE1-AS in HCT-8 cells treated with LPS or PDTC in HCT-8 cells following *C. parvum* infection at 24 hpi by using RT-qPCR. Three independent experiments were performed. Statistical analysis was conducted by using a non-parametric *t*-test. *P < 0.05, **P < 0.01.

The mRNA levels of BACE1-AS were significantly downregulated in HCT-8 cells during *C. parvum* infection from 8 h post-infection (pi) to 48 hpi by RT-qPCR analysis, with the lowest expression level at 8 hpi (Fig. 1A). A previous study indicated that the NF-κB signaling pathway could affect the expression of miR-181d in HCT-8 cells during *C. parvum* infection (31). To investigate whether the abnormal expression of BACE1-AS induced by *C. parvum* infection was induced by the NF-κB signaling pathway or not, HCT-8 cells were treated with lipopolysaccharide (LPS) (5 µg/mL) and pyrrolidine dithiocarbamate (PDTC) (1.643 µg/mL, an inhibitor for NF-κB signaling pathway), respectively, and the results indicated LPS stimulation also induced the downregulation of BACE1-AS in HCT-8 cells, while PDTC treatment reversed the downregulation of BACE1-AS in HCT-8 cells infected with *C. parvum* at 24 hpi (Fig. 1B). Further studies found both *T. gondii* and *N. caninum* could not significantly alter the mRNA levels of BACE1-AS in HCT-8 cells at 24 hpi, indicating that the downregulation of BACE1-AS in HCT-8 cells was likely specifically induced by *C. parvum* (Fig. S3). Taken together, *C. parvum* infection likely induces the downregulation of BACE1-AS by activating the NF-κB signaling pathway.

### Overexpression of BACE1-AS delays the propagation of *C. parvum* in HCT-8 cells

**Fig 2:**
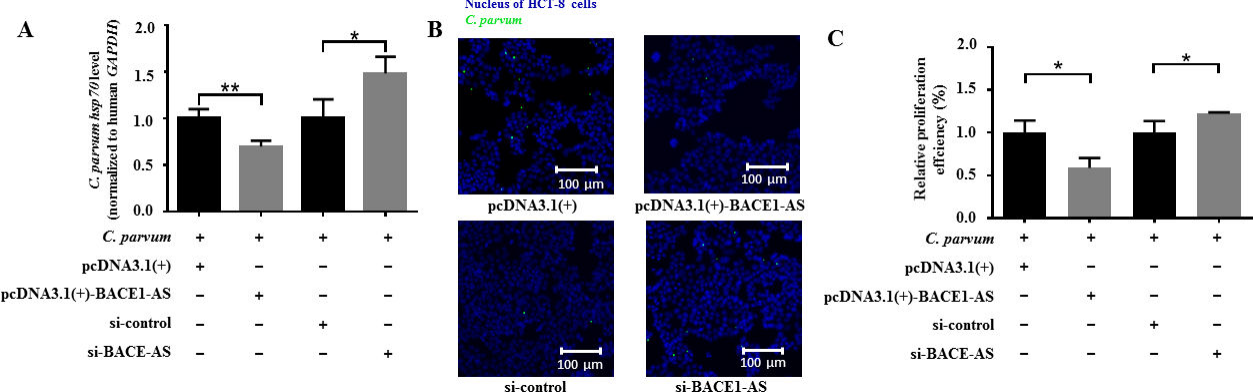
Effect of BACE1-AS on the propagation of *C. parvum* in HCT-8 cells. (A) Effect of BACE1-AS on the mRNA levels of the *C. parvum hsp70* gene in HCT-8 cells at 24 hpi. pcDNA3.1(+), pcDNA3.1(+)-BACE1-AS, si-control and si-BACE1-AS represent empty overexpression vector, overexpression vector of BACE1-AS, universal control siRNA and specific siRNA of BACE1-AS, respectively. Results of RT-qPCR were normalized to human *GAPDH*, and the relative mRNA level was calculated by using the 2^−ΔΔCt^ method. (B) Effect of BACE1-AS on the propagation efficiency of *C. parvum* in HCT-8 cells at 24 hpi. Individual color channels have been adjusted according to the journal image integrity rules. (C) Statistical analysis of the propagation efficiency of *C. parvum* in HCT-8 cells at 24 hpi. A total of 50 randomly selected visual fields of each slide were captured for quantification analysis. The propagation efficiency of *C. parvum* was calculated by counting the average percentage of FITC-labeled *C. parvum* to DAPI-labeled HCT-8 cells in each microscope field. The relative propagation efficiency of *C. parvum* was analyzed by using the 2^−ΔΔCt^ method. Three independent experiments were performed. Statistical analysis was conducted by using a non-parametric *t* test. *P < 0.05, **P < 0.01. Data underlying this figure can be found in Supplementary Material S1.

To explore the function of BACE1-AS during *C. parvum* infection, the propagation of *C. parvum* in HCT-8 cells was detected by both RT-qPCR and IFA. RT-qPCR found that overexpression of BACE1-AS significantly decreased the mRNA levels of the *C. parvum hsp70* gene, while a contrary result was identified for the knockdown of BACE1-AS (Fig. 2A). Simultaneously, IFA results indicated that overexpression of BACE1-AS significantly decreased the propagation efficiency of *C. parvum* in HCT-8 cells at 24 hpi, while the contrary result was identified for the knockdown of BACE1-AS (Fig. 2B and C). These results suggest that BACE1-AS can inhibit the propagation of *C. parvum* in HCT-8 cells.

### BACE1-AS enhances the expression of *IRF3* by sponging miR-6805-5p in HCT-8 cells during *C. parvum* infection

**Fig 3:**
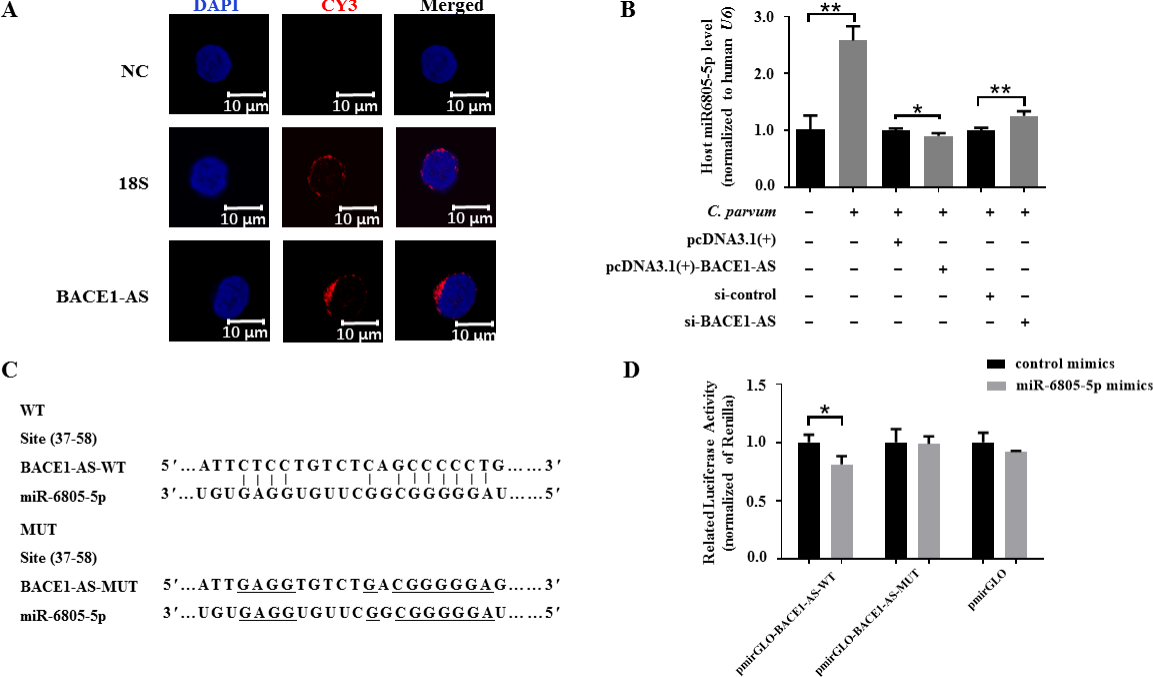
Sponging relationship between BACE1-AS and miR-6805-5p in HCT-8 cells during *C. parvum* infection. (A) Subcellular location of BACE1-AS in HCT-8 cells. HCT-8 cells were seeded on a coverslip and were infected with *C. parvum* for 24 h and then applied for FISH analysis of BACE1-AS using probes conjugated with cy3, with *18S* localized in the cytoplasm as a control. The nucleus was visualized by DAPI staining. (B) The mRNA levels of miR-6805-5p in HCT-8 cells transfected with pcDNA3.1(+)-BACE1-AS plasmid or si-BACE1-AS following *C. parvum* infection at 24 hpi by using RT-qPCR. (C) Putative binding site between BACE1-AS and miR-6805-5p predicted by LncBook. (D) The luciferase activity in HCT-8 cells co-transfected with miR-6805-5p mimics and pmirGLO-BACE1-AS-WT or pmirGLO-BACE1-AS-MUT. Statistical analysis was conducted by using a non-parametric *t*-test. *P < 0.05, **P < 0.01. WT, wild type.

Location of lncRNAs in cells is closely related to their mechanisms (32–34). To determine the potential mechanism of BACE1-AS, the location of BACE1-AS in HCT-8 cells was identified by using the FISH assay, and the results indicated that BACE1-AS was majorly located outside of the nucleus of HCT-8 cells (Fig. 3A), reflecting that BACE1-AS likely functioned by sponging miRNAs, which has been reported in human diseases, particularly neurodegenerative diseases and cancers (35–39).

To explore miRNAs potentially targeted by BACE1-AS, a total of 205 targeted miRNAs of BACE1-AS were identified by using LncBook. Among them, 12 highly matched miRNAs (miR-6805-5p, miR-541-3p, miR-143-3p, miR-762, miR-5088-3p, miR-654-3p, miR-12120-3p, miR-5582-5p, miR-6798-5p, miR-6803-5p, miR-6851-5p, and miR-296-5p) were selected for further analysis. Further study indicated that seven miRNAs (miR-6805-5p, miR-296-5p, miR-762, miR-6851-5p, miR-143-3p, miR-654-3p, and miR-541-3p) were significantly upregulated in HCT-8 cells infected with *C. parvum* at 24 hpi (Fig. S4A). However, only the mRNA level of miR-6805-5p was negatively regulated by BACE1-AS at 24 hpi (Fig. S4B and C). Transfection with pcDNA3.1(+)-BACE1-AS significantly reversed the upregulated expression of miR-6805-5p in HCT-8 cells infected with *C. parvum* at 24 hpi, while the contrary result was found in the group transfected with si-BACE1-AS (Fig. 3B). Additionally, the dual-luciferase reporter assay showed that miR-6805-5p mimics could significantly decrease the luciferase activity of pmirGLO-BACE1-AS-WT (*P* < 0.05) but not that of pmirGLO-BACE1-AS-MUT (Fig. 3C and D). These results suggest that miR-6805-5p is a direct target for BACE1-AS in HCT-8 cells during *C. parvum* infection.

**Fig 4:**
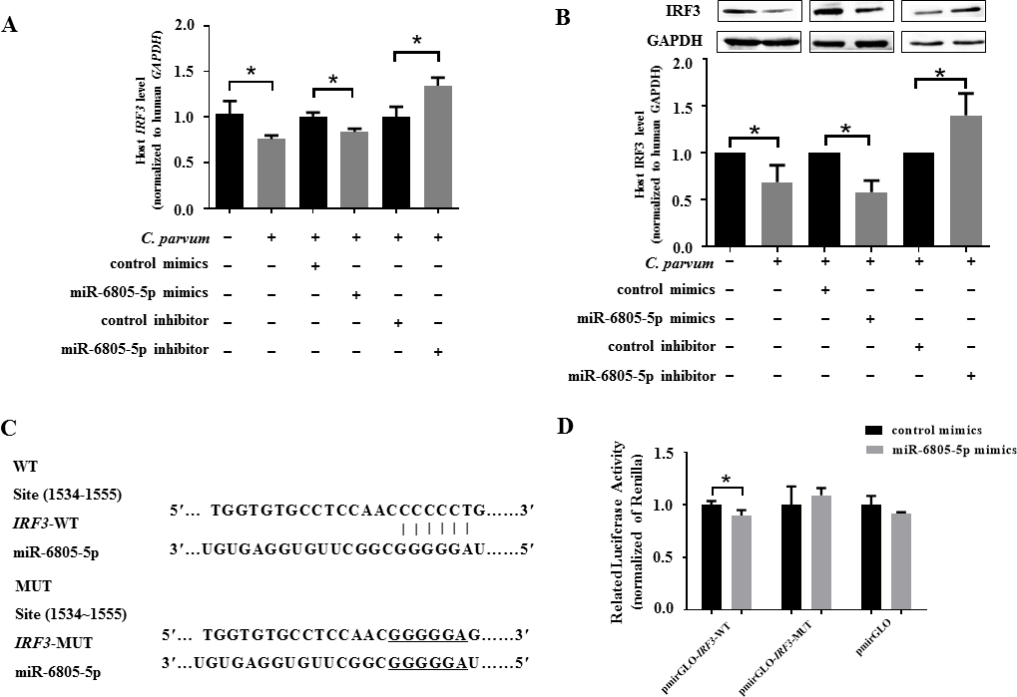
Target relationship between miR-6805-5p and *IRF3* in HCT-8 cells following *C. parvum* infection. The mRNA (A) and protein (B) levels of *IRF3* in HCT-8 cells transfected with miR-6805-5p mimics or inhibitor following *C. parvum* infection at 24 hpi by RT-qPCR and Western blot. ImageJ was used to calculate the protein level normalized to GAPDH by densitometry. (C) Putative binding site between the 3′-UTR of *IRF3* and miR-6805-5p predicted by miRWalk. (D) The luciferase activity in HCT-8 cells co-transfected with miR-6805-5p mimics and pmirGLO-*IRF3*-WT or pmirGLO-*IRF3*-MUT. Three independent experiments were performed. Statistical analysis was conducted by using a non-parametric *t-*test. *P < 0.05.

To explore mRNAs potentially targeted by miR-6805-5p, a total of 10 highly matched targets of miR-6805-5p, namely, *interferon regulatory factor 3* (*IRF3*), *caudal type homeobox 2* (*CDX2*), *growth arrest-specific 7* (*GAS7*), *high mobility group AT-hook 1* (*HMGA1*), *TNF superfamily member 13* (*TNFSF13*), *interferon regulatory factor 9* (*IRF9*), *autophagy-related 12* (*ATG12*), *centrosomal protein 164* (*CEP164*), *mitogen-activated protein kinase 2* (*MAPK2*), and *phosphatase and tensin homolog* (*PTEN*), were predicted by using miRWalk and applied for further analysis. Among them, five genes (*CDX2*, *PTEN*, *IRF9*, *IRF3*, and *CEP164*) were significantly downregulated in HCT-8 cells infected with *C. parvum* at 24 hpi (Fig. S5A). However, only the mRNA level of *IRF3* was negatively affected by miR-6805-5p at 24 hpi (Fig. S5B and C). Transfection with miR-6805-5p mimics significantly downregulated the mRNA and protein levels of *IRF3* at 24 hpi during *C. parvum* infection, while the contrary results were found for the miR-6805-5p inhibitor (Fig. 4A and B). Meanwhile, the dual-luciferase reporter assay showed that miR-6805-5p mimics could significantly decrease the luciferase activity of pmirGLO-*IRF3*-WT (*P* < 0.05) but not of pmirGLO-*IRF3*-MUT (Fig. 4C and D). These results suggest that *IRF3* is a direct target for miR-6805-5p in HCT-8 cells during *C. parvum* infection.

**Fig 5:**
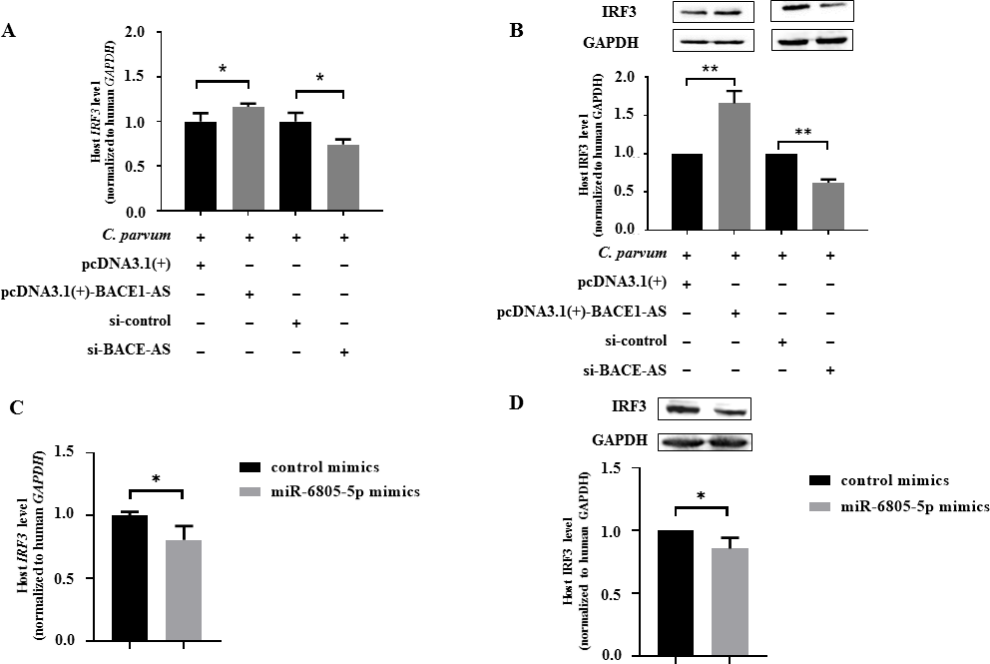
Effect of BACE1-AS on the expression of *IRF3* in *C. parvum*-infected HCT-8 cells via sponging miR-6805-5p. The mRNA (A) and protein (B) levels of *IRF3* in HCT-8 cells transfected with pcDNA3.1(+)-BACE1-AS plasmid or si-BACE1-AS following *C. parvum* infection at 24 hpi by RT-qPCR and Western blot. The mRNA (C) or protein (D) levels of *IRF3* in HCT-8 cells co-transfected with pcDNA3.1(+)-BACE1-AS plasmid and miR-6805-5p mimics following *C. parvum* infection at 24 hpi by RT-qPCR and Western blot, respectively. Three independent experiments were performed. Statistical analysis was conducted by using a non-parametric *t*-test. *P < 0.05, **P < 0.01.

Interestingly, overexpression of BACE1-AS significantly promoted the mRNA and protein expression level of the *IRF3* gene in HCT-8 cells during *C. parvum* infection at 24 hpi, while the contrary result was found for knockdown of BACE1-AS (Fig. 5A and B). Furthermore, co-transfection results found that miR-6805-5p mimics reversed the upregulation effect of BACE1-AS on *IRF3* at both mRNA and protein levels (Fig. 5C and D). Taken together, BACE1-AS can regulate the expression of *IRF3* in HCT-8 cells during *C. parvum* infection via sponging miR-6805-5p.

### BACE1-AS/miR-6805-5p/IRF3 axis delays the propagation of *C. parvum* in HCT-8 cells

**Fig 6:**
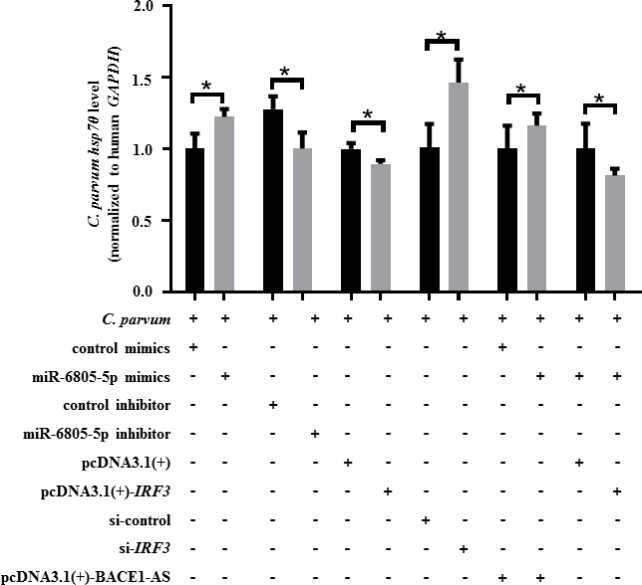
Effect of the BACE1-AS/miR-6805-5p/IRF3 axis on the expression of *C. parvum hsp70* gene in HCT-8 cells. HCT-8 cells transfected with miR-6805-5p mimics, miR-6805-5p inhibitor, pcDNA3.1(+)-*IRF3,* si-*IRF3,* pcDNA3.1(+)-BACE1-AS and miR-6805-5p mimics mixture, or miR-6805-5p mimics and pcDNA3.1(+)-*IRF3* mixture were infected with *C. parvum* at 24 hpi. The mRNA levels of *C. parvum hsp70* gene were detected by RT-qPCR. Three independent experiments were performed. Statistical analysis was conducted by using a non-parametric *t* test. *P < 0.05.

**Fig 7:**
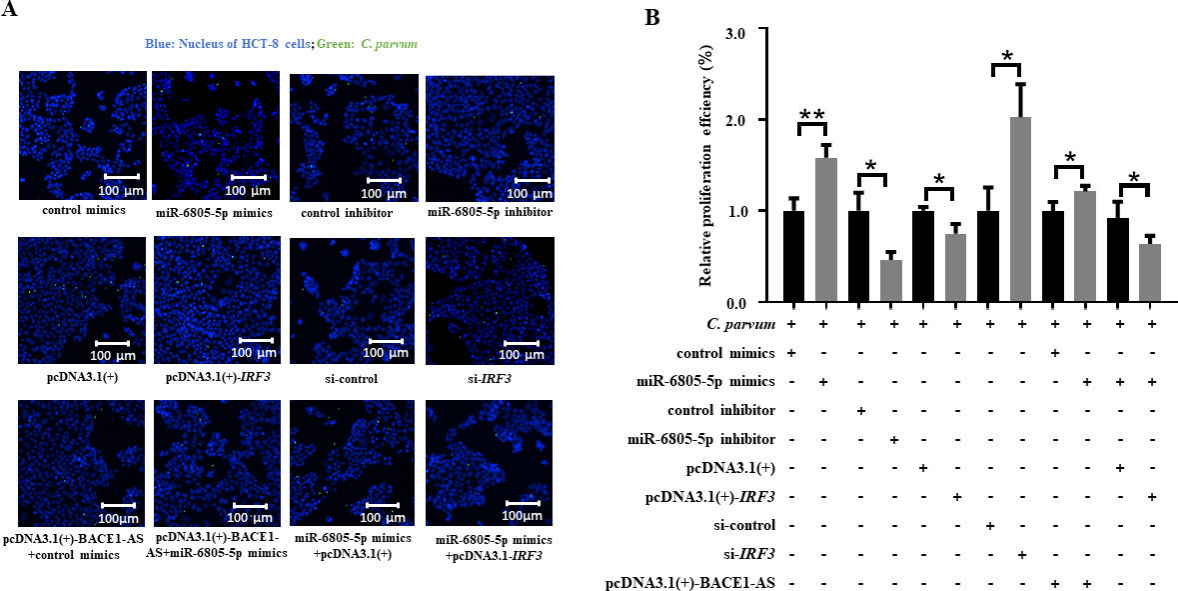
Effect of the BACE1-AS/miR-6805-5p/IRF3 axis on the propagation efficiency of *C. parvum* in HCT-8 cells. HCT-8 cells transfected with miR-6805-5p mimics, miR-6805-5p inhibitor, pcDNA3.1(+)-*IRF3,* si-*IRF3,* pcDNA3.1(+)-BACE1-AS and miR-6805-5p mimics mixture, or miR-6805-5p mimics and pcDNA3.1(+)-*IRF3* mixture were infected with *C. parvum* at 24 hpi. The propagation efficiency of *C. parvum* in HCT-8 cells at 24 hpi was detected by indirect immunofluorescence analysis (A). Statistical analysis of the propagation efficiency of *C. parvum* in HCT-8 cells at 24 hpi was conducted by non-parametric *t-*test (B). Three independent experiments were performed. *P < 0.05, **P < 0.01. Data underlying this figure can be found in Supplementary Material S1.

To explore the function of the BACE1-AS/miR-6805-5p/IRF3 axis during *C. parvum* infection, the expression level of *C. parvum hsp70* was detected by using RT-qPCR, and the propagation rate of *C. parvum* in HCT-8 cells was detected by IFA. RT-qPCR results showed that miR-6805-5p mimics or si-*IRF3* significantly promoted the mRNA level of the *C. parvum hsp70* gene in HCT-8 cells at 24 hpi, while the contrary result was identified for miR-6805-5p inhibitor or pcDNA3.1(+)-*IRF3* (Fig. 6). Additionally, co-transfection results indicated that miR-6805-5p mimics significantly reversed the downregulation of the *C. parvum hsp70* gene expression in HCT-8 cells induced by pcDNA3.1(+)-BACE1-AS (Fig. 6), and pcDNA3.1(+)-*IRF3* significantly reversed the upregulation of *C. parvum hsp70* gene expression in HCT-8 cells regulated by miR-6805-5p mimics (Fig. 6). Meanwhile, IFA results indicated that miR-6805-5p mimics or si-*IRF3* significantly promoted the propagation efficiency of *C. parvum* in HCT-8 cells at 24 hpi, while the contrary result was identified for miR-6805-5p inhibitor or pcDNA3.1(+)-*IRF3* (Fig. 7). Additionally, co-transfection results indicated that miR-6805-5p mimics significantly reversed the downregulation of the propagation efficiency of *C. parvum* in HCT-8 cells induced by pcDNA3.1(+)-BACE1-AS (Fig. 7), and pcDNA3.1(+)-*IRF3* reversed the upregulation of the propagation efficiency of *C. parvum* in HCT-8 cells regulated by miR-6805-5p mimics (Fig. 7). These results suggest that the BACE1-AS/miR-6805-5p/IRF3 axis can delay the propagation of *C. parvum* in HCT-8 cells.

### BACE1-AS/miR-6805-5p/IRF3 axis inhibits BCL2-mediated apoptosis of HCT-8 cells during *C. parvum* infection

**Fig 8:**
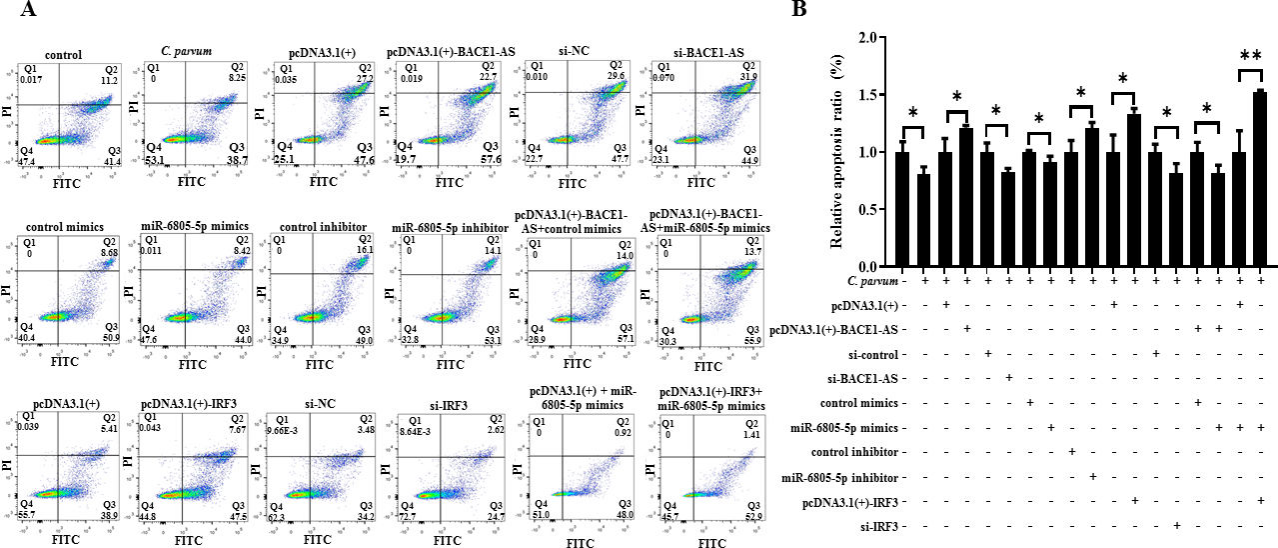
Effect of the BACE1-AS/miR-6805-5p/IRF3 axis on the apoptosis of HCT-8 cells infected with *C. parvum*. HCT-8 cells transfected with pcDNA3.1(+)-BACE1-AS, si-BACE1-AS, miR-6805-5p mimics, miR-6805-5p inhibitor, pcDNA3.1(+)-*IRF3,* si-*IRF3,* pcDNA3.1(+)-BACE1-AS and miR-6805-5p mimics mixture, or miR-6805-5p mimics and pcDNA3.1(+)-*IRF3* mixture were infected with *C. parvum* at 24 hpi. The cell apoptosis of HCT-8 cells at 24 hpi was detected by flow cytometry (A). The relative apoptosis ratio of HCT-8 cells at 24 hpi (B). Annexin V labeled with green fluorescent probe YF488 and PI exhibited red fluorescence under laser excitation and was used in flow cytometry analysis with the gate value of 10^3^–10^4^ based on the recommendation from flow cytometer instructions and technicians. Three independent experiments were performed. Statistical analysis was conducted by using a non-parametric *t-*test. *P < 0.05, **P < 0.01.

*C. parvum* infection could inhibit cell apoptosis at early infection (2–24 hpi), and the contrary results have been found at late infection (>24 hpi) (40–43). In this study, the effect of the BACE1-AS/miR-6805-5p/IRF3 axis on cell apoptosis during *C. parvum* infection was explored by using flow cytometry analysis. *C. parvum* infection significantly inhibited the apoptosis level of HCT-8 cells at 24 hpi (Fig. 8). Interestingly, pcDNA3.1(+)-BACE1-AS, miR-6805-5p inhibitor, and pcDNA3.1(+)-*IRF3* significantly promoted the apoptosis of HCT-8 cells, while the opposite results were found for si-BACE1-AS, miR-6805-5p mimics, and si-*IRF3* (Fig. 8). In addition, co-transfection results found that miR-6805-5p mimics significantly reversed the upregulation of the apoptosis levels induced by pcDNA3.1(+)-BACE1-AS, and pcDNA3.1(+)-*IRF3* significantly reversed the downregulation of apoptosis levels induced by miR-6805-5p mimics (Fig. 8). Taken together, the BACE1-AS/miR-6805-5p/IRF3 axis can inhibit the apoptosis level of HCT-8 cells during *C. parvum* infection.

**Fig 9:**
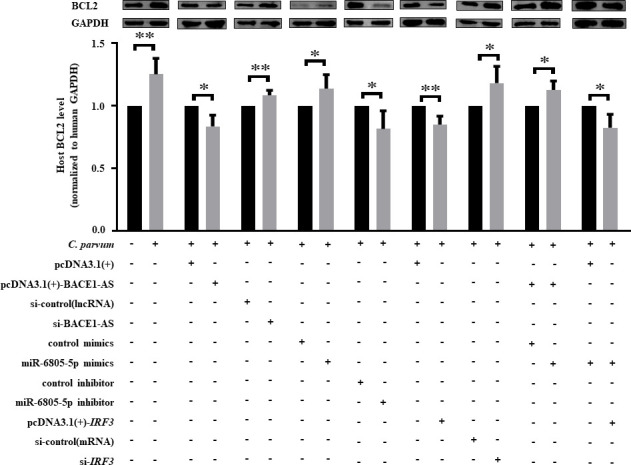
Effect of the BACE1-AS/miR-6805-5p/IRF3 axis on the expression of BCL2 in HCT-8 cells during *C. parvum* infection. HCT-8 cells transfected with pcDNA3.1(+)-BACE1-AS, si-BACE1-AS, miR-6805-5p mimics, miR-6805-5p inhibitor, pcDNA3.1(+)-*IRF3*, si-*IRF3*, [pcDNA3.1(+)-BACE1-AS] and miR-6805-5p mimics mixture, or miR-6805-5p mimics and pcDNA3.1(+)-*IRF3* mixture were infected with *C. parvum* at 24 hpi. The protein expression levels of BCL2 were analyzed by Western blot. Three independent experiments were performed. Statistical analysis was conducted by using a non-parametric *t* test. *P < 0.05, **P < 0.01. Data underlying this figure can be found in Supplementary Material S2.

Previous studies have found that *C. parvum* can regulate cell apoptosis to promote its intracellular survival by both extrinsic and intrinsic pathways (41–44). As an immunoregulatory factor in host defense against pathogens, IRF3 has been recognized as a pro-apoptotic factor to promote cell apoptosis through the interaction between ubiquitinated IRF3 and apoptosis factor Bax (45–47). Our group found that *C. parvum* infection significantly upregulated the protein level of BCL-2, cleaved caspase-3, and BAX (28), and the present study found that transfection of pcDNA3.1(+)-BACE1-AS significantly (*P* < 0.05) downregulated the protein level of BCL2 during *C. parvum* infection but had no effect on the expression of cleaved caspase-3 and BAX (Fig. S6). Furthermore, transfection of si-BACE1-AS significantly (*P* < 0.05) upregulated the expression of BCL2 during *C. parvum* infection (Fig. 9). Meanwhile, miR-6805-5p mimics significantly upregulated the expression of BCL2 during *C. parvum* infection, while miR-6805-5p inhibitor showed the opposite effect on the expression of BCL2 (Fig. 9). pcDNA3.1(+)-*IRF3* significantly downregulated the expression of BCL2 during *C. parvum* infection, while the opposite was found for si-*IRF3* (Fig. 9). Interestingly, co-transfection results found that miR-6805-5p mimics significantly reversed the downregulated expression of BCL2 induced by pcDNA3.1(+)-BACE1-AS, and pcDNA3.1(+)-*IRF3* significantly reversed the upregulated expression of BCL2 induced by miR-6805-5p mimics (Fig. 9). In summary, BACE1-AS can target the miR-6805-5p/IRF3 axis to inhibit BCL2-mediated apoptosis of HCT-8 cells during *C. parvum* infection.

## DISCUSSION

Increasing evidence indicates that lncRNAs can be functional in physiological and pathological processes through regulating the expression of downstream genes at transcriptional and post-transcriptional levels (15, 34, 48–50). *Cryptosporidium* infection can significantly alter the expression profile of host lncRNAs (19, 20, 49). Further studies indicate that several significantly upregulated lncRNAs during *C. parvum* infection can regulate intestinal anti-*Cryptosporidium* defense to delay the intracellular survival of parasites (24, 25, 27). The present study identified a significantly downregulated host lncRNA BACE1-AS, which could affect the propagation of *C. parvum in vitro*.

Previous studies have found that infection with *C. parvum*, *T. gondii,* and *N. caninum* could activate the NF-κB signaling pathway of host cells (31, 51, 52), but only *C. parvum* infection specifically downregulated the expression of BACE1-AS in host cells by triggering the NF-κB signaling pathway in the present study. Those three pathogens have different tissue tropisms, and *T. gondii* can invade most cell types, while *C. parvum* and *N. caninum* majorly infect intestinal epithelial cells and uterine epithelial cells, respectively. Furthermore, infections with *C. parvum*, *T. gondii,* and *N. caninum* lead to divergent changes in the expression profiles of lncRNAs in infected cells (19, 22, 53). In the present study, human intestinal epithelial cells (HCT-8) were used to establish an *in vitro* infection model, and only *C. parvum* infection caused the downregulation of BACE1-AS in this cell line.

LncRNAs are a class of RNA molecules lacking an obvious reading frame and coding ability (54). They primarily exert regulatory functions by participating in chromosome inactivation, epigenetic regulation, and transcriptional regulation (15, 17, 55). Among these roles, the extensively investigated one is the capacity of lncRNAs to modulate gene expression through sequestering specific microRNAs via their miRNA response elements, thereby regulating host immune responses and contributing to antiviral or antiparasitic effects (56, 57). For example, the tumor suppressor lncRNA F630028O10Rik inhibited lung cancer angiogenesis by regulating the expression of miR-223-3p (58). LncRNA MARL enhanced the expression of mitochondrial antiviral-signaling proteins (MAVs) to inhibit the replication of *Siniperca chuatsi* rhabdovirus by sponging miR-122 (59). Chronic hepatitis B virus induced the upregulation of lncRNA n335586, and the overexpression of n335586 promoted the expression of host CKMT1A by competitive binding to miR-924 to promote the migration and invasion of hepatocellular carcinoma cells (60).

As a non-coding antisense transcript of BACE1, lncRNA BACE1-AS plays important roles in neurodegenerative diseases and cancers and can also be used as a diagnostic marker of Alzheimer’s disease (35, 36, 61–63). Recent studies have found that BACE1-AS could use its microRNA response elements (MREs) to sponge specific miRNAs and then regulate the expression of target genes to play a crucial role in the occurrence and development of a variety of diseases (35, 36, 62). For example, silence of BACE1-AS mitigated neuronal damage by regulating autophagy through the miR-214-3p/ATG5 signaling axis (36). BACE1-AS enhanced the invasion and metastasis of hepatocellular cancer cells by mediating the miR-377-3p/CELF1 axis (62). Downregulation of BACE1-AS inhibited the activation of iNOS in the substantial nigra by upregulating miR-34b-5p and downregulating BACE1, and then improved oxidative stress damage of rats with Parkinson’s disease (63). Berberine protected neuronal cells from partial damage of amyloid β_25–35_ by the BACE1-AS/miR-132-3p axis (64). In this study, BACE1-AS was majorly distributed outside of the nucleus of HCT-8 cells, and further studies indicated that BACE1-AS could target the miR-6805-5p/IRF3 axis to affect the propagation of *C. parvum in vitro*.

Apoptosis is an important immune means of the hosts against parasitic infection and has been extensively studied in *C. parvum* infection (65–67). *C. parvum* could induce cell apoptosis by regulating the expression of apoptosis-related factors in host cells, such as inducing downregulation of IFI27, and further regulating the apoptosis of HCT-8 cells to inhibit the infection of *C. parvum* to host cells through the tumor necrosis factor (TNF)-related apoptosis inducing ligand (TRAIL)-dependent pathway (68). *In vitro* infection of HCT-8 cells with *C. parvum* could induce survivin expression of the antiapoptotic protein family gene to inhibit apoptosis and promote the development of *C. parvum* (69). Meanwhile, miR-4521 further affected BCL2-mediated endogenous apoptosis through targeted regulation of *FOXM1*, thus promoting the propagation of *C. parvum* in HCT-8 cells (28). MiR-3976 regulated cell apoptosis and parasite burden in HCT-8 cells by targeting BCL2A1 following *C. parvum* infection (70). *C. parvum* induced the downregulation of IFI27 via miR-942-5p-mediated translational suppression (68). These pieces of evidence suggested that *C. parvum* infection could regulate the apoptosis of host cells and thus affect its intracellular survival. In this study, *C. parvum* infection regulated the BACE1-AS/miR-6805-5p/IRF3 axis to affect cell apoptosis, which could expand our understanding of the regulation of cell apoptosis by *Cryptosporidium*.

**Fig 10:**
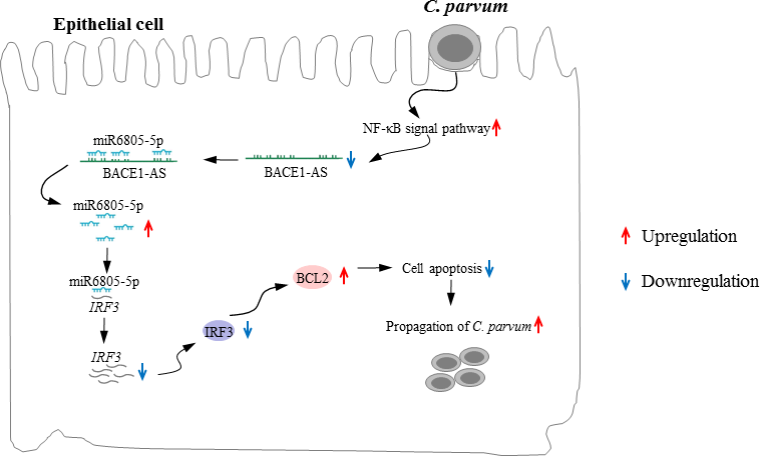
Model of BACE1-AS in the regulation of the propagation of *C. parvum*. BACE1-AS is likely downregulated by the activation of the NF-κB signaling pathway in HCT-8 cells following *C. parvum* infection. BACE1-AS can sponge miR6805-5p to upregulate the level of *IRF3*, and then downregulation of BACE1-AS leads to downregulation of *IRF3*. Downregulated *IRF3* can upregulate BCL2 to decrease the apoptosis level of cells, thus promoting the propagation of *C. parvum*.

As a key link in innate immunity, IRF3 plays a wide range of biological functions in many diseases and cellular processes, such as tumorigenesis, metabolic reprogramming, antiviral infection, and occurrence and development of autoimmune diseases (71–75). Among them, the most widely studied function is to regulate type I interferon in anti-infection immunity; e.g., African swine fever virus E120R protein interacted with host cell IRF3 to block the production of beta interferon and then inhibit the host’s antiviral immunity (71). Meanwhile, IRF3 played an important role in autophagy, apoptosis, and other cellular processes (76, 77). LPS-treated mice induced the activation of the STING-IRF3 pathway, which then activated NLRP3, leading to apoptosis and inflammation (78). In non-alcoholic fatty liver disease, the activated STING-IRF3 pathway also promoted hepatocyte apoptosis and induced metabolic disorders (76). In addition, previous studies revealed that IRF3 could be recognized as an apoptogenic factor (47, 76, 79). Further analysis in the present study indicated that IRF3 could be regulated by the BACE1-AS/miR-6805-5p axis to affect the expression of BCL2 to promote the apoptosis of HCT-8 cells and then inhibited the propagation of *C. parvum in vitro*, which contributed to our understanding of the function of IRF3 in the interaction between hosts and *Cryptosporidium* (Fig. 10).

In conclusion, *C. parvum* infection downregulated the expression of host BACE1-AS by activating the NF-кB signal pathway. The downregulated BACE1-AS affected BCL2-induced cell apoptosis by targeting the miR-6805-5p/IRF3 axis to promote its propagation in HCT-8 cells. Results of the present study can enrich our knowledge on the understanding of the pathogenic mechanism of *C. parvum* and provide potential novel targets for the development of vaccines and drugs for cryptosporidiosis.

## SUPPLEMENTAL MATERIAL

Supplementary Material 1 spectrum.02022-24-s0001.pdf Representative IFA images of Fig. 2 and Fig. 7.

Supplementary Material 2 spectrum.02022-24-s0002.pdf Full blots of Fig. 9 and Fig. S6.

Fig. S1 spectrum.02022-24-s0003.TIF The effects of overexpression and inhibition on BACE1-AS (A) and miR-6805-5p (B) in HCT‑8 cells.

Fig. S2 spectrum.02022-24-s0004.TIF The effects of overexpression and inhibition on *IRF3* at mRNA (A) and protein (B) levels in HCT‑8 cells.

Fig. S3 spectrum.02022-24-s0005.TIF Expression of BACE1-AS in HCT-8 cells during infections of *T. gondii* (A) or *N. caninum* (B).

Fig. S4 spectrum.02022-24-s0006.TIF Expression of partial target miRNAs of BACE1-AS in HCT-8 cells.

Fig. S5 spectrum.02022-24-s0007.TIF Expression of partial target genes of miR-6805-5p in HCT-8 cells.

Fig. S6 spectrum.02022-24-s0008.tif Expression of proteins related to apoptosis in HCT-8 cells transfected with pcDNA3.1(+)-BACE1-AS following *C. parvum* infection at 24 hpi.

Table S1 spectrum.02022-24-s0009.docx Specific sequences of si-RNAs, mimic, and inhibitors used in this study.

ASM does not own the copyrights to Supplemental Material that may be linked to, or accessed through, an article. The authors have granted ASM a non-exclusive, world-wide license to publish the Supplemental Material files. Please contact the corresponding author directly for reuse.
