# Supplementary material for: LncRNA BACE1-AS delays the propagation of Cryptosporidium parvum through regulating cell apoptosis by targeting the miR-6805-5p/IRF3 axis
Source: Microbiol Spectr. 2025 Jun 9;13(7):e02022-24. doi: 10.1128/spectrum.02022-24 (PMC12211009; doi:10.1128/spectrum.02022-24)
Supplement: Supplementary Material 1 — Representative IFA images of Fig. 2 and Fig. 7. [file spectrum.02022-24-s0001.pdf]

Supplementary Material 1

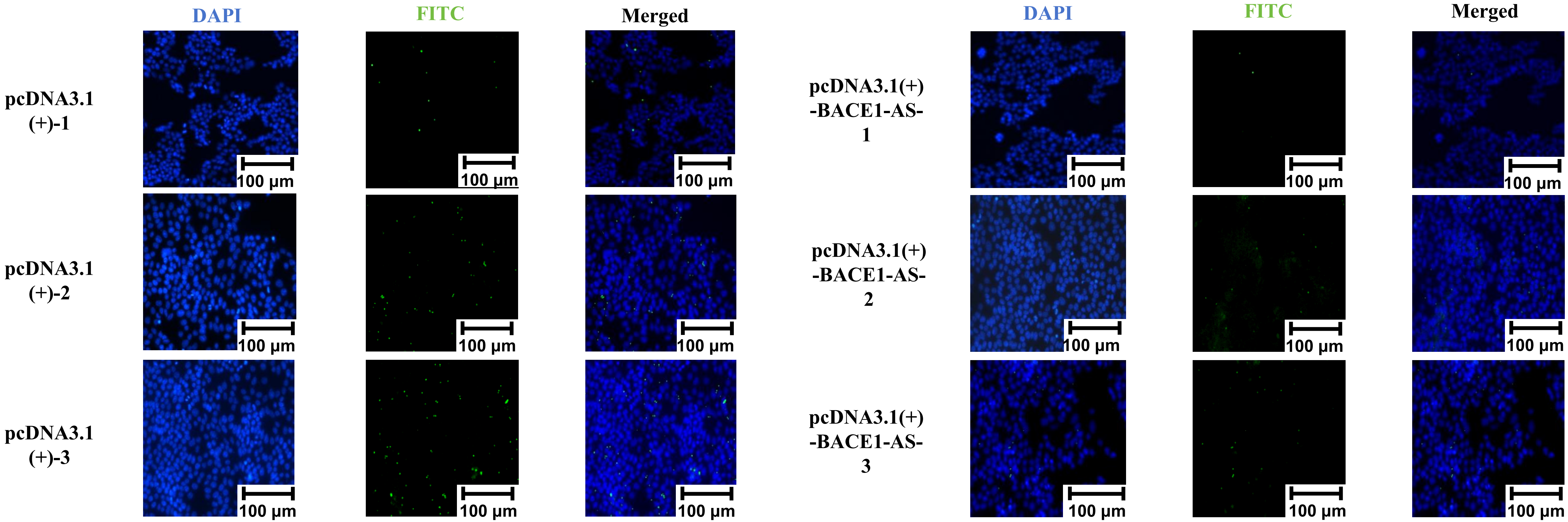

Supplementary Material 1 – Representative IFA images of Fig. 2

Supplementary Material 1

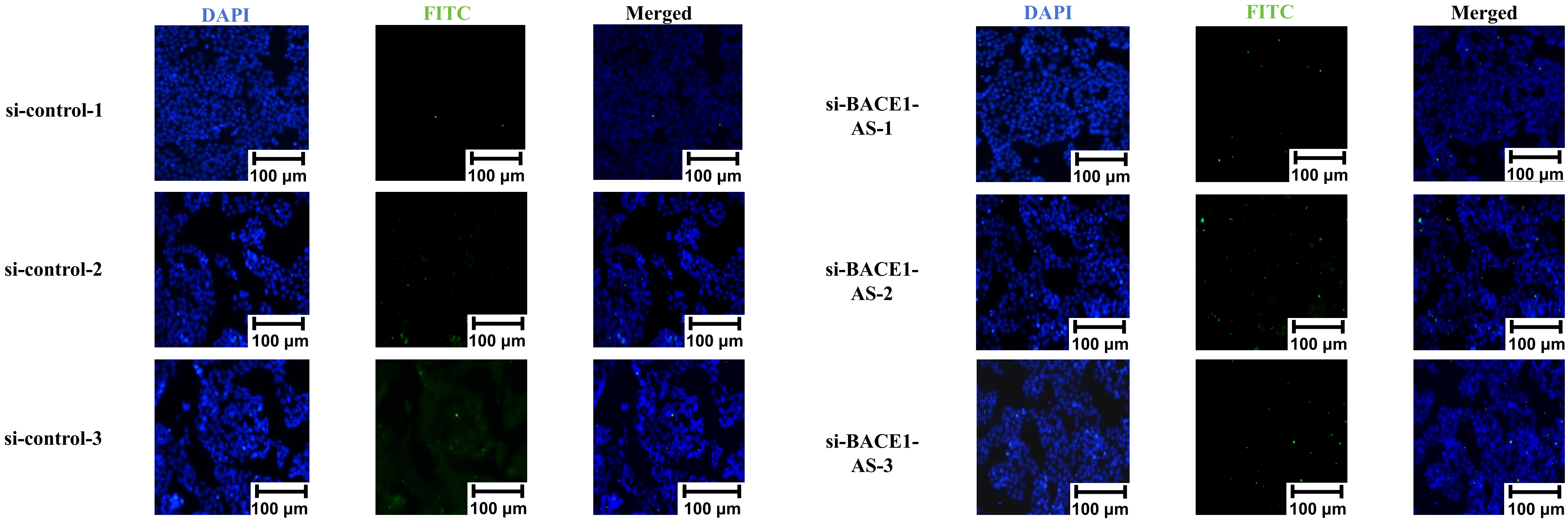

Supplementary Material 1 – Representative IFA images of Fig. 2

Supplementary Material 1

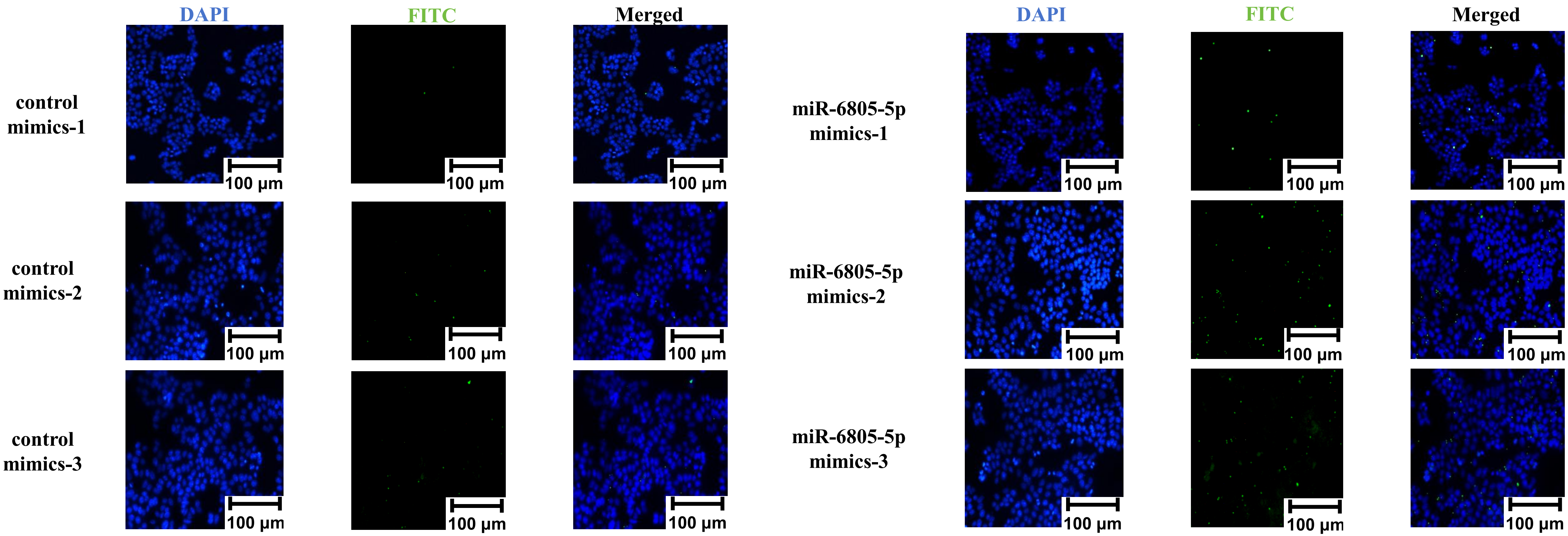

Supplementary Material 1 – Representative IFA images of Fig. 7

Supplementary Material 1

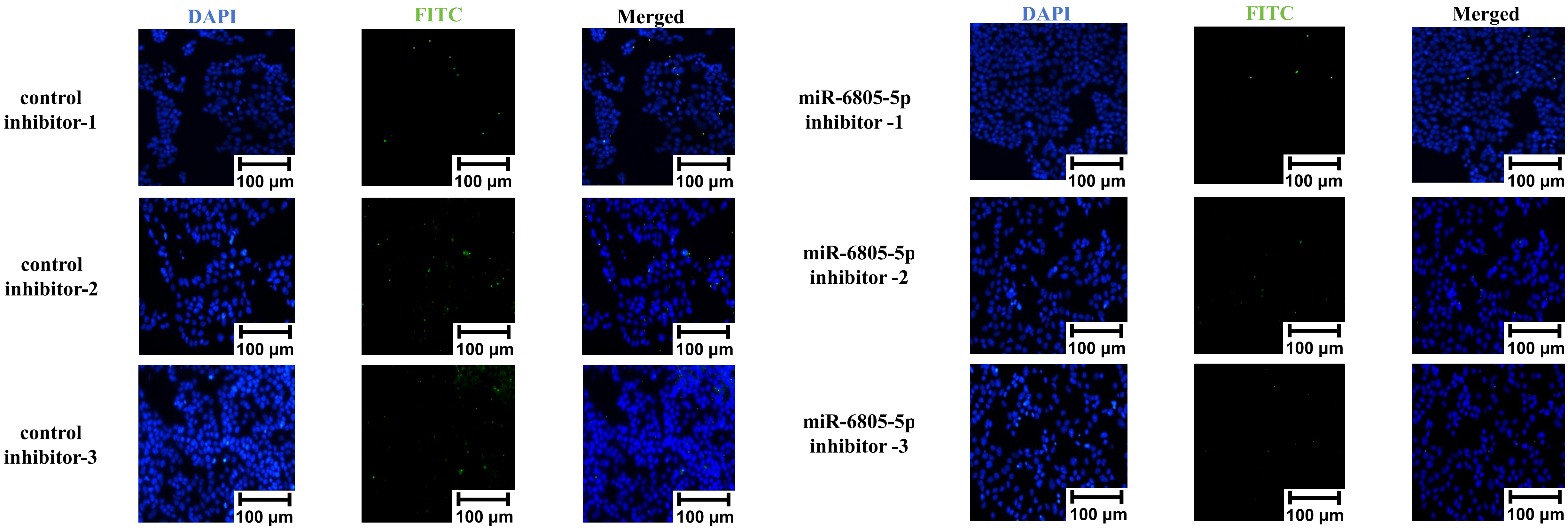

Supplementary Material 1 – Representative IFA images of Fig. 7

Supplementary Material 1

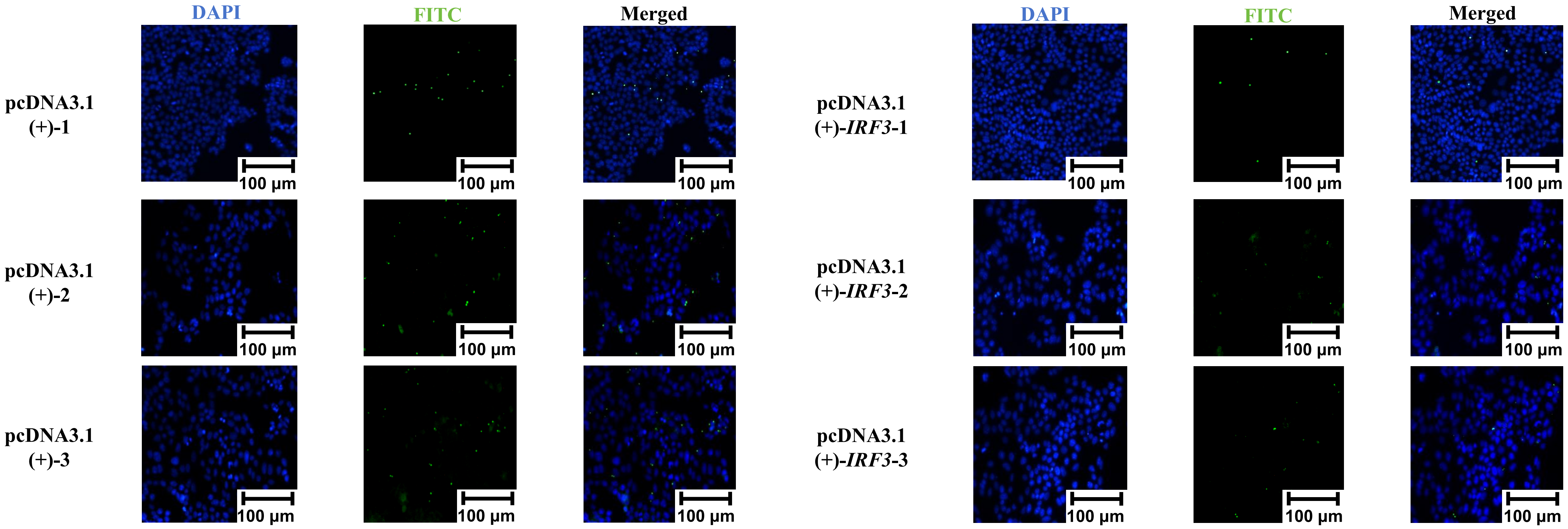

Supplementary Material 1 – Representative IFA images of Fig. 7

Supplementary Material 1

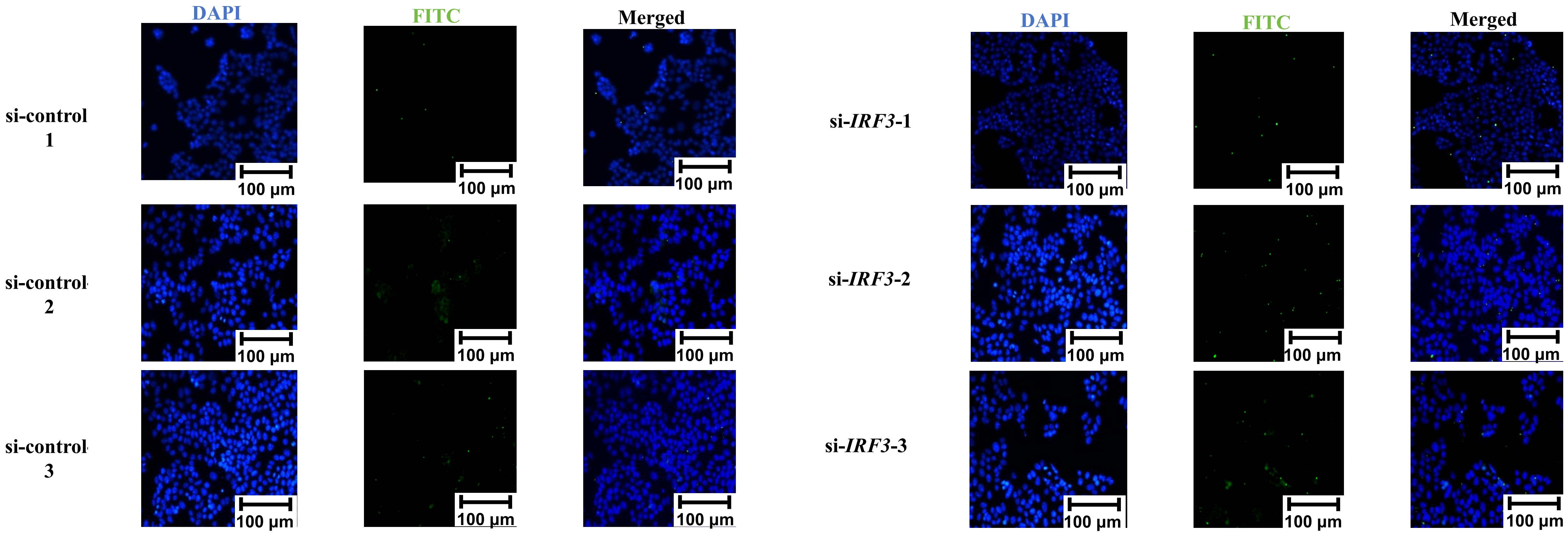

Supplementary Material 1 – Representative IFA images of Fig. 7

Supplementary Material 1

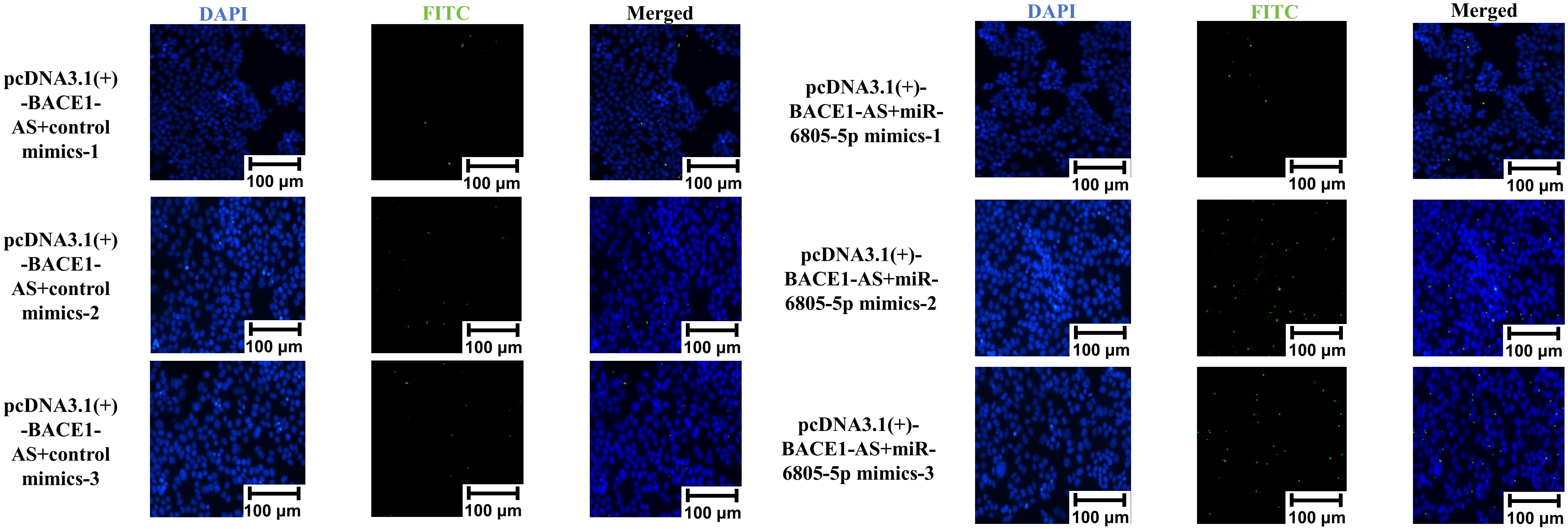

Supplementary Material 1 – Representative IFA images of Fig. 7

Supplementary Material 1

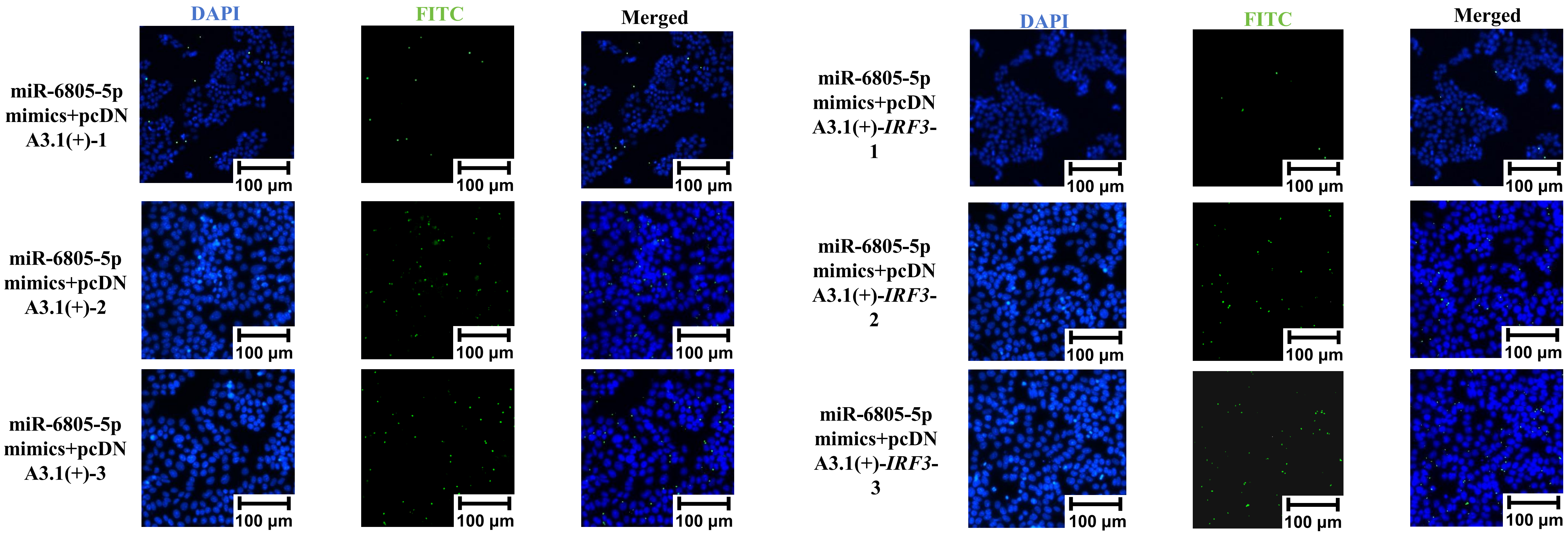

Supplementary Material 1 – Representative IFA images of Fig. 7
