# Supplementary material for: LncRNA BACE1-AS delays the propagation of Cryptosporidium parvum through regulating cell apoptosis by targeting the miR-6805-5p/IRF3 axis
Source: Microbiol Spectr. 2025 Jun 9;13(7):e02022-24. doi: 10.1128/spectrum.02022-24 (PMC12211009; doi:10.1128/spectrum.02022-24)
Supplement: Supplementary Material 2 — Full blots of Fig. 9 and Fig. S6. [file spectrum.02022-24-s0002.pdf]

Supplementary Material 2

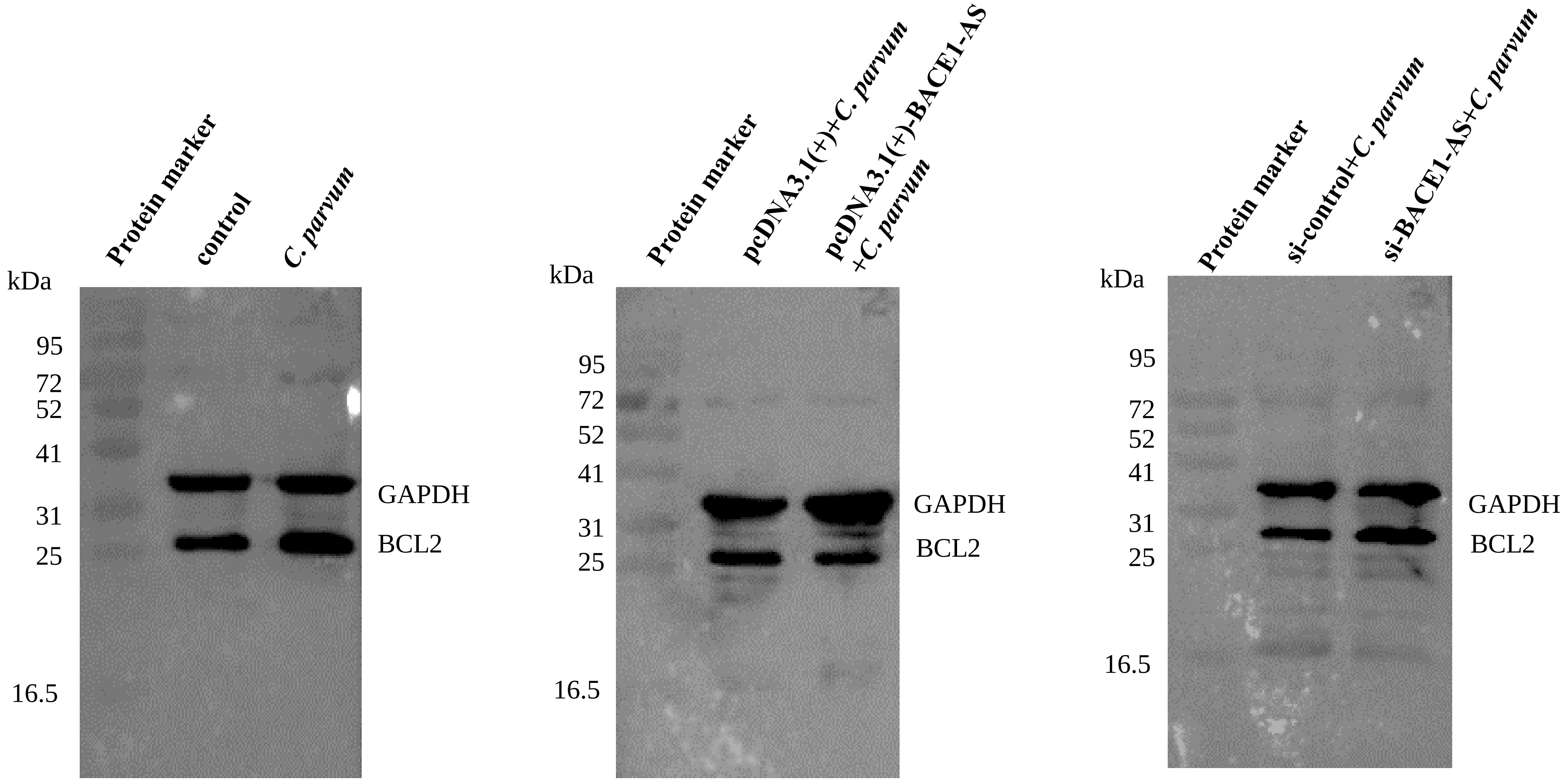

Supplementary Material 2 – Full blots of Fig. 9

Supplementary Material 2

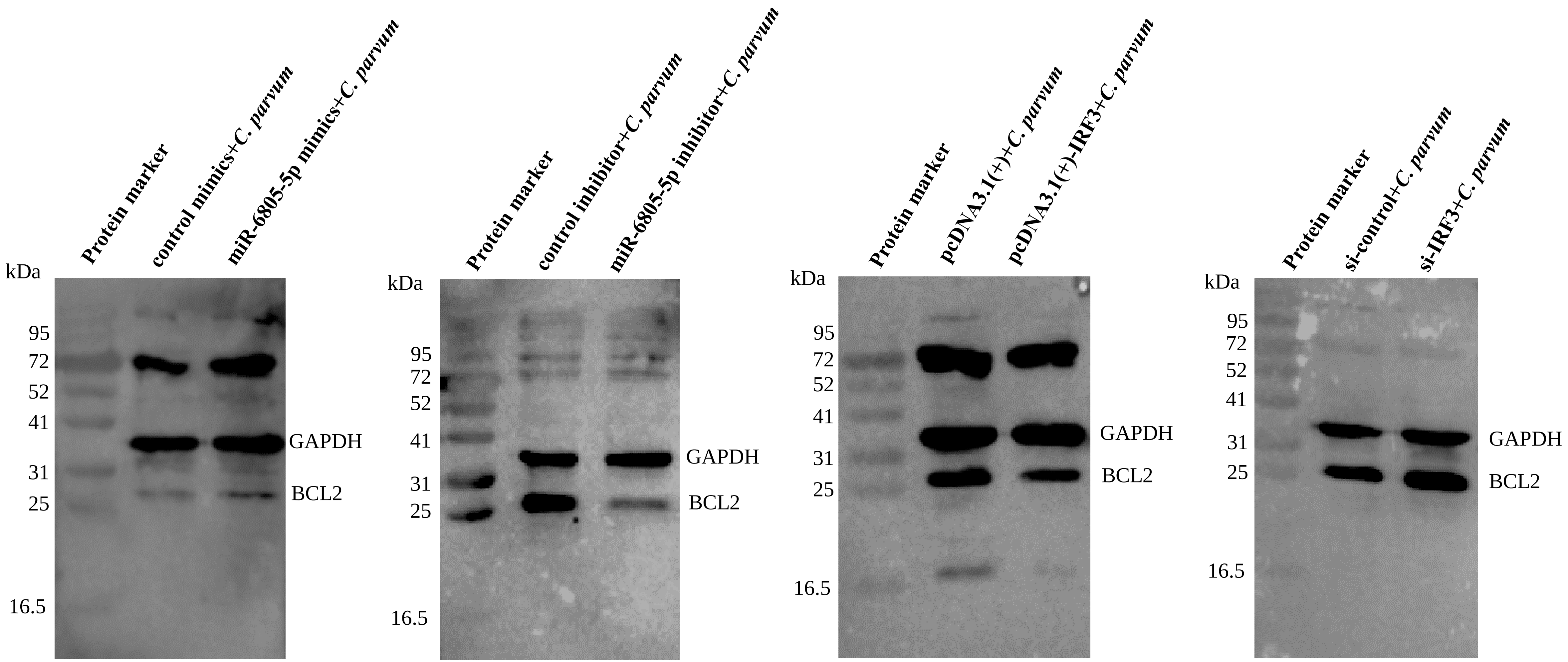

Supplementary Material 2 – Full blots of Fig. 9

Supplementary Material 2

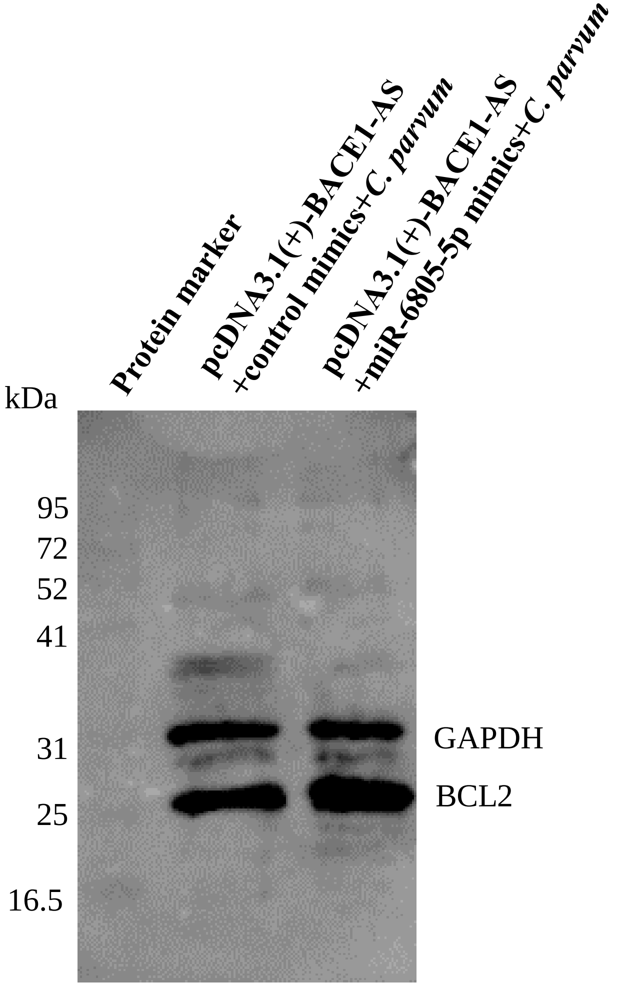

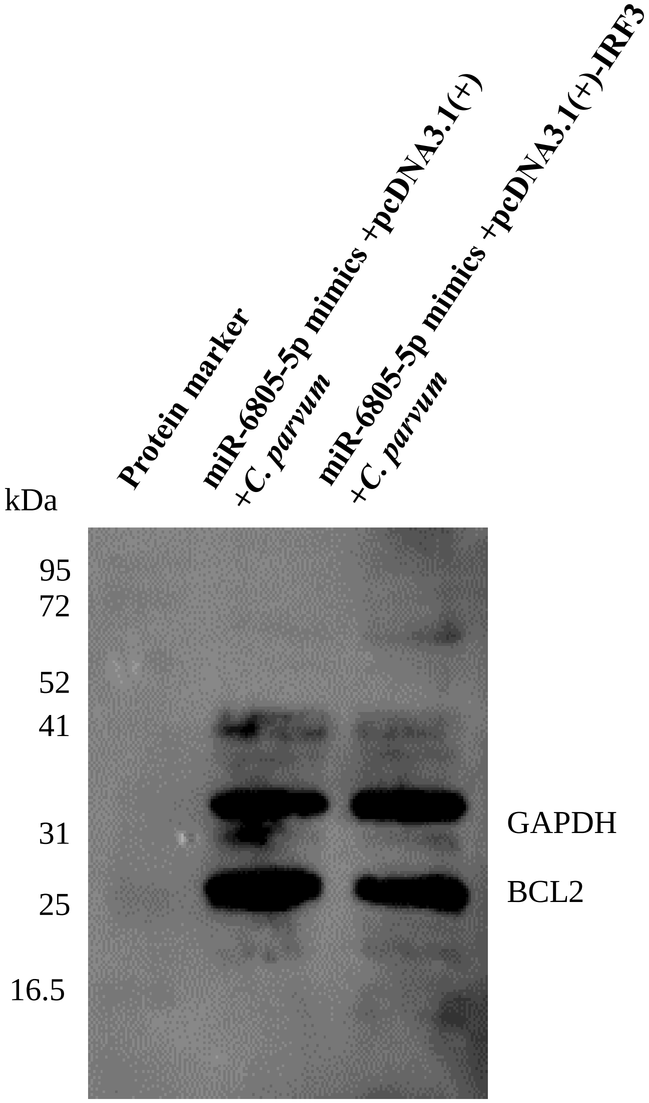

Supplementary Material 2 – Full blots of Fig. 9

Supplementary Material 2

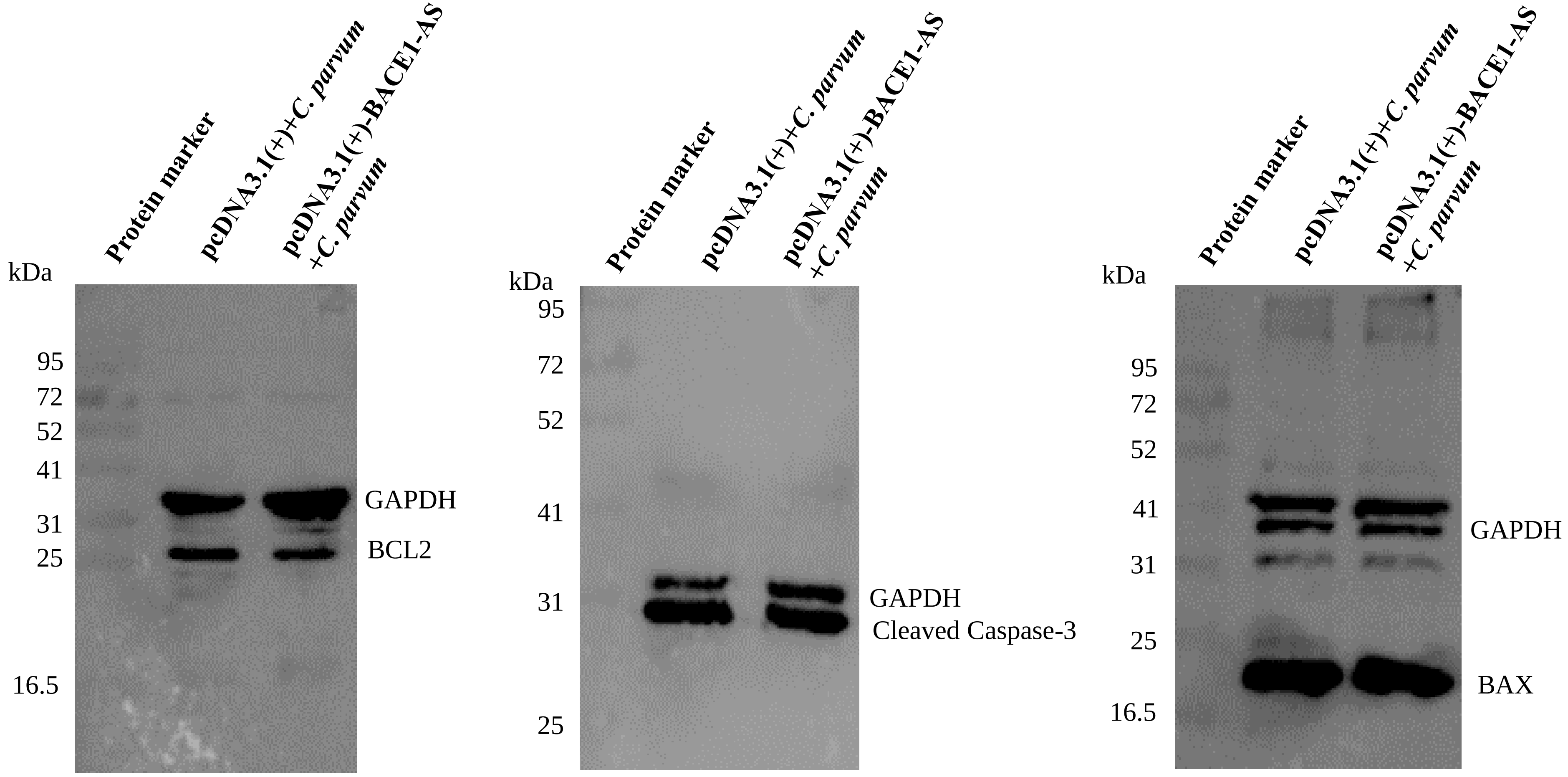

Supplementary Material 2 – Full blots of Fig. S6
