# Supplementary figures and images for: LncRNA BACE1-AS delays the propagation of Cryptosporidium parvum through regulating cell apoptosis by targeting the miR-6805-5p/IRF3 axis
Source: Microbiol Spectr. 2025 Jun 9;13(7):e02022-24. doi: 10.1128/spectrum.02022-24 (PMC12211009; doi:10.1128/spectrum.02022-24)

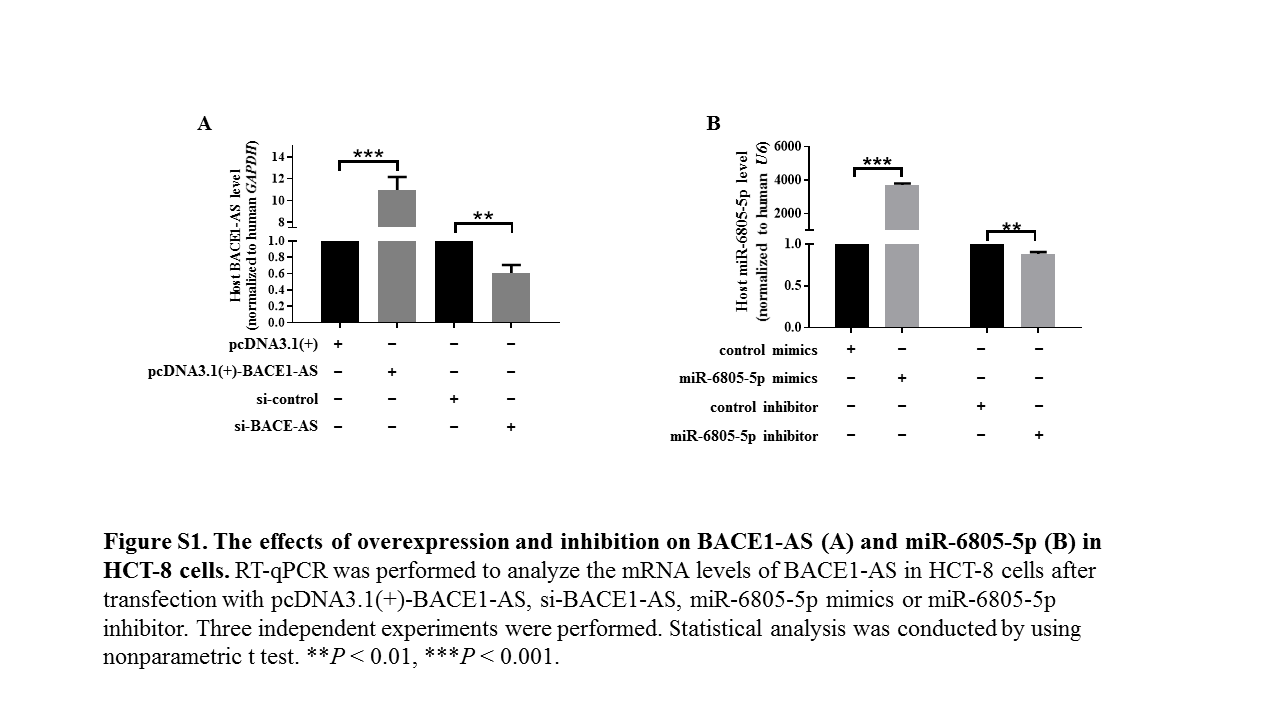

Supplement: Fig. S1 — The effects of overexpression and inhibition on BACE1-AS (A) and miR-6805-5p (B) in HCT‑8 cells. [file spectrum.02022-24-s0003.tif]

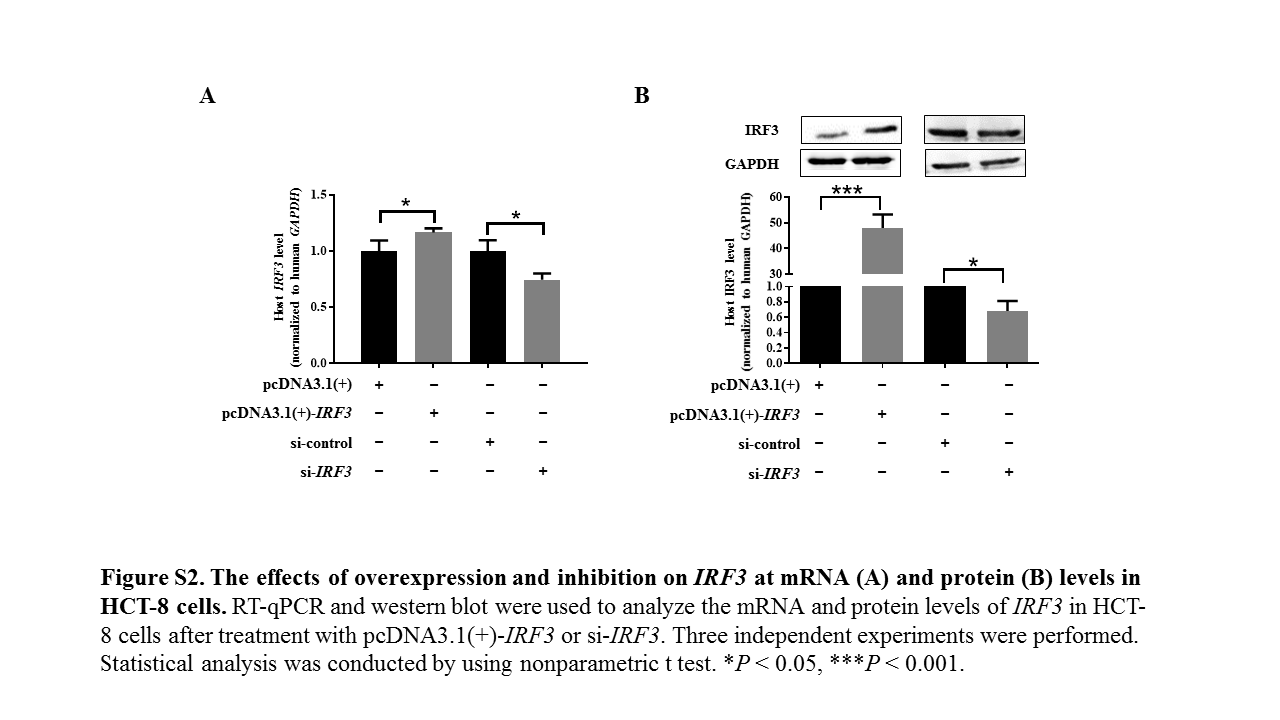

Supplement: Fig. S2 — The effects of overexpression and inhibition on IRF3 at mRNA (A) and protein (B) levels in HCT‑8 cells. [file spectrum.02022-24-s0004.tif]

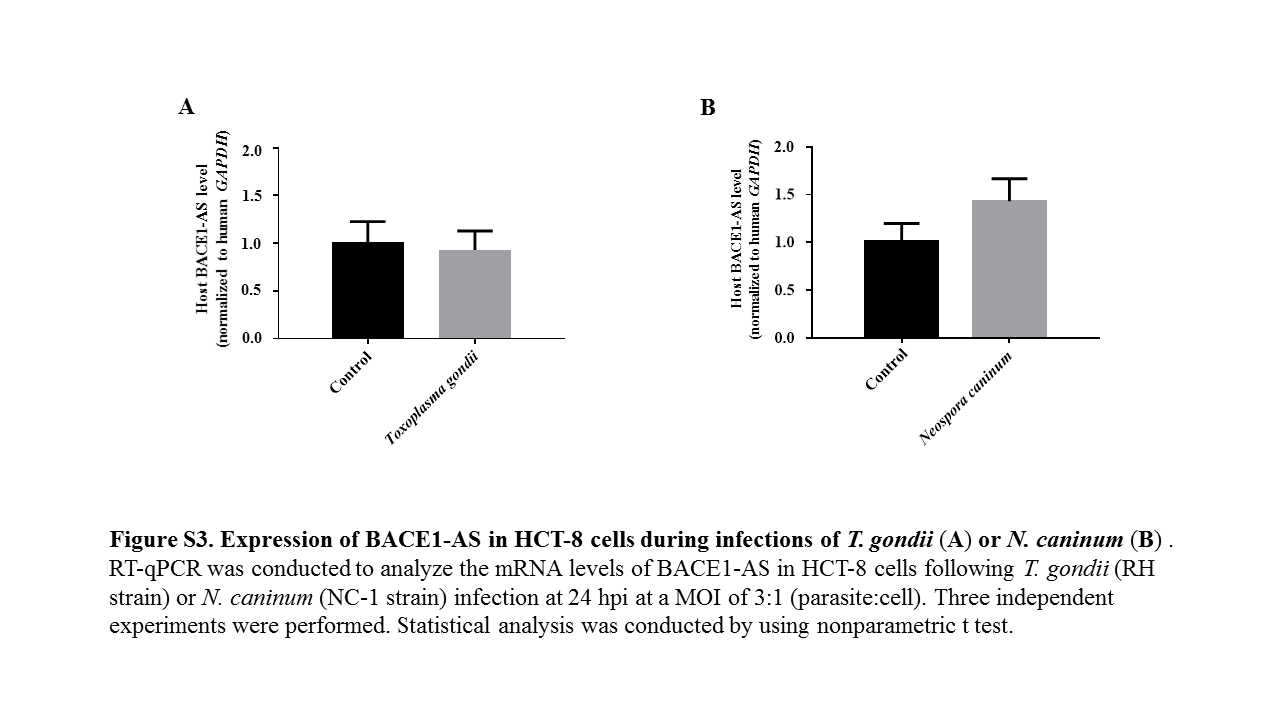

Supplement: Fig. S3 — Expression of BACE1-AS in HCT-8 cells during infections of T. gondii (A) or N. caninum (B). [file spectrum.02022-24-s0005.tif]

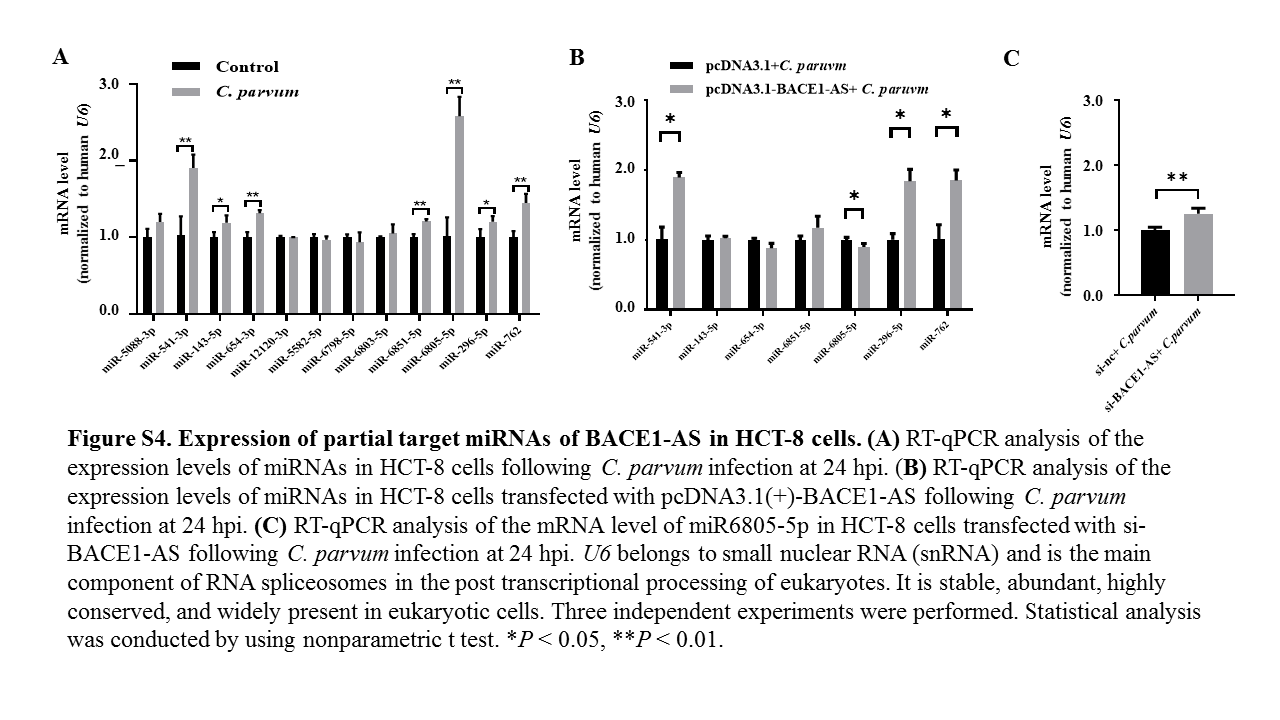

Supplement: Fig. S4 — Expression of partial target miRNAs of BACE1-AS in HCT-8 cells. [file spectrum.02022-24-s0006.tif]

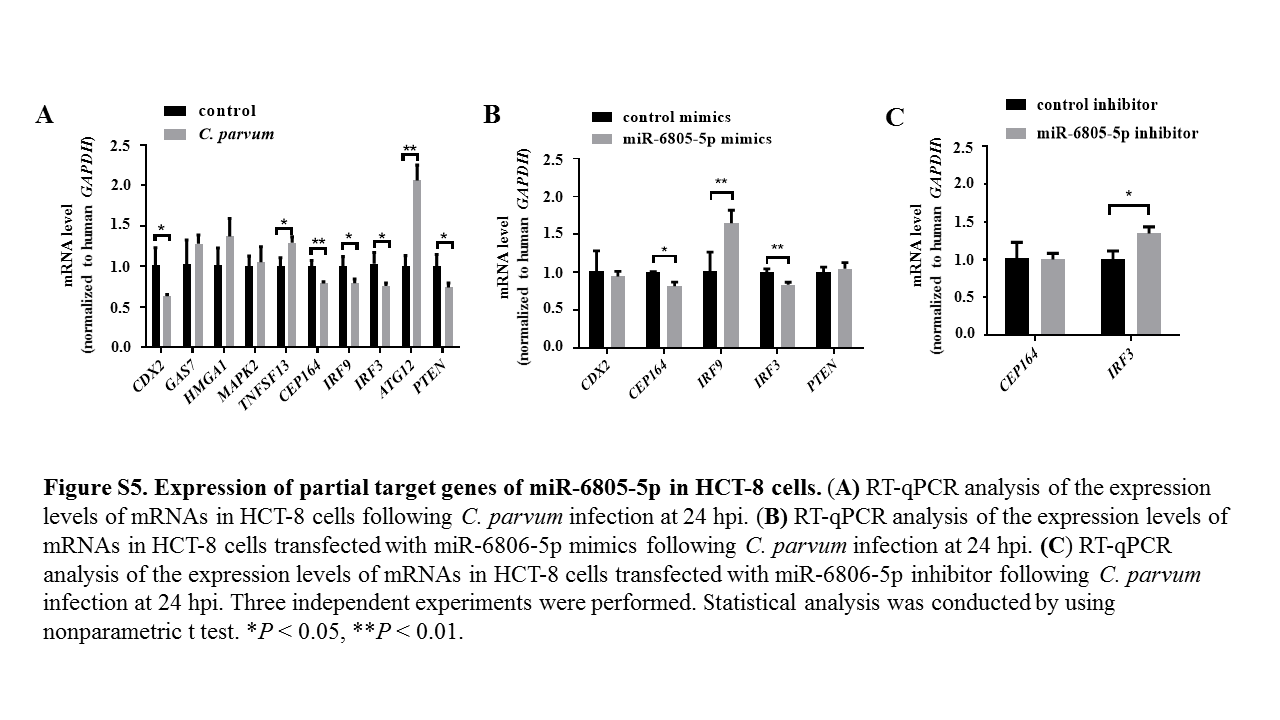

Supplement: Fig. S5 — Expression of partial target genes of miR-6805-5p in HCT-8 cells. [file spectrum.02022-24-s0007.tif]

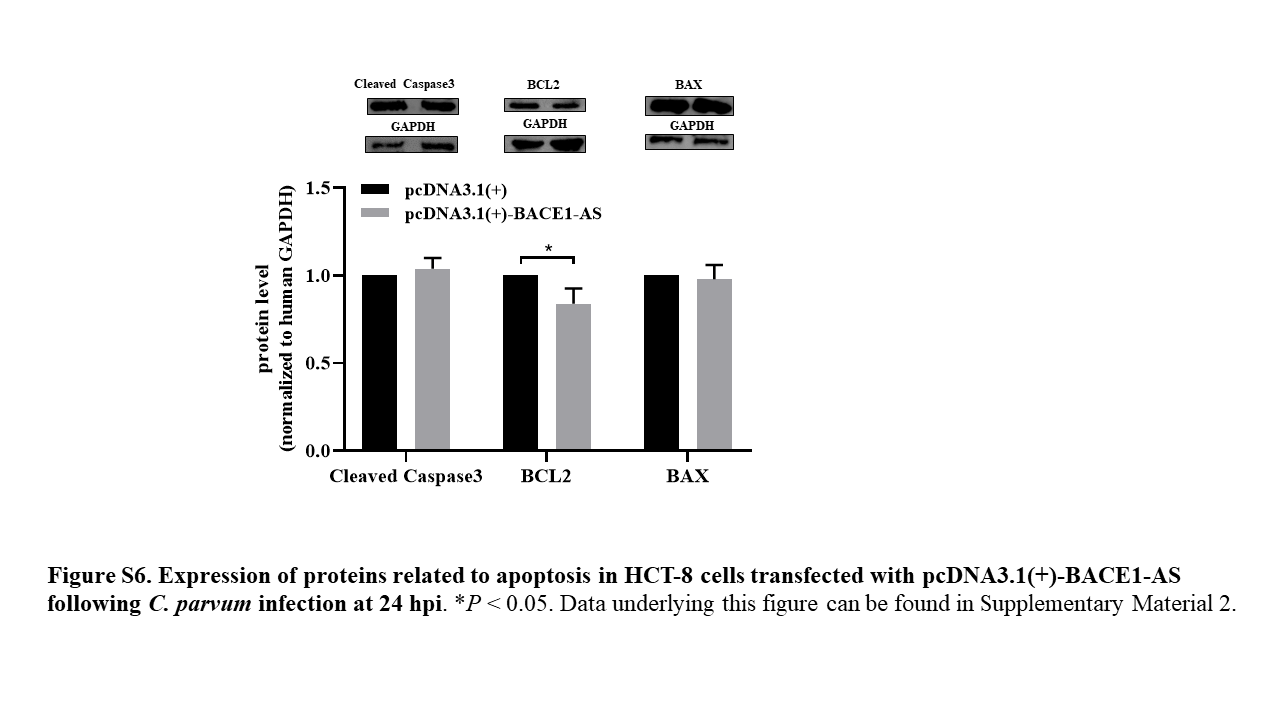

Supplement: Fig. S6 — Expression of proteins related to apoptosis in HCT-8 cells transfected with pcDNA3.1(+)-BACE1-AS following C. parvum infection at 24 hpi. [file spectrum.02022-24-s0008.tif]
