# Supplementary material for: LncRNA BACE1-AS delays the propagation of Cryptosporidium parvum through regulating cell apoptosis by targeting the miR-6805-5p/IRF3 axis
Source: Microbiol Spectr. 2025 Jun 9;13(7):e02022-24. doi: 10.1128/spectrum.02022-24 (PMC12211009; doi:10.1128/spectrum.02022-24)
Supplement: Table S1 — Specific sequences of si-RNAs, mimic, and inhibitors used in this study. [file spectrum.02022-24-s0009.docx]

**Table S1** Specific sequences of si-RNAs, mimic and inhibitors used in this study.

| **Name** |  | **Sequences (5′-3′)** |
| --- | --- | --- |
| BACE1-AS | si-RNA | GCAGGTAGTCTCCAAACTT |
| miR-6805-5p | mimics | UAGGGGGCGGCUUGUGGAGUGU |
|  | inhibitor | ACACUCCACAAGCCGCCCCCUA |
| *IRF3* | si-RNA | CCCUUCAUUGUAGAUCUGATT |
